# SGK1 upregulation in GFAP^+^ neurons in the frontal association cortex protects against neuronal apoptosis after spinal cord injury

**DOI:** 10.1038/s41419-025-07542-y

**Published:** 2025-04-02

**Authors:** Anbiao Wu, Guang Yang, Genyu Liu, Jiyan Zhang

**Affiliations:** Beijing Institute of Basic Medical Sciences, Beijing, China

**Keywords:** Spinal cord diseases, Cell death in the nervous system

## Abstract

Spinal cord injury (SCI) casts devastating and long-lasting impacts on the well-being of patients. Cognitive deficits and emotional disorders are common in individuals with SCI, yet the underlying mechanisms are not completely understood. Astrogliosis and glial scar formation occur during the subacute phase post-injury, playing complicated roles in remyelination and neurite regrowth. Therefore, we constructed a GFAP-IRES-Venus-AkaLuc knock-in mouse model for the corresponding studies. Surprisingly, complete spinal cord transection (SCT) surgery led to earlier and more prominent augmentation of bioluminescence in the brain than in the spinal cord. Bulk RNA sequencing revealed the activation of apoptotic signaling and the upregulation of serum and glucocorticoid-regulated kinase 1 (SGK1). The pattern of GFAP signals changed throughout the brain after SCT, as indicated by tissue clearing and immunostaining. Specifically, GFAP signals were intensified in the frontal association cortex (FrA), an encephalic region involved in associative learning and recognition memory processes. Further exploration unraveled that intensified GFAP signals in the FrA were attributed to apoptotic neurons with SGK1 upregulation, which was induced by sustained high glucocorticoid levels after SCT. The introduction of SGK1 silencing vectors confirmed that SGK upregulation in these FrA neurons exerted anti-apoptotic effects through NRF2/HO-1 signaling. In addition, SGK1 knockdown in FrA neurons aggravated the post-SCI depressive-like behaviors. Thus, ectopic SGK1 expression designated for limbic neurons could serve as a promising therapeutic target for the future development of treatments for spinal cord injuries.

## Introduction

Spinal cord injury (SCI) leads to significantly reduced lifespans as well as chronic decreases in living qualities, such as motor and sensory dysfunction, neuralgia, and secondary neuroinflammation elsewhere [1]. The natural progression of spinal cord injuries has generally been characterized into three distinct pathological phases [2, 3]. In the acute phase (0 to 2 days after injury), the primary mechanical injuries of the spinal cord initiate hemorrhage, ischemia, neuronal death, demyelination and inflammatory cell infiltration; then, in the subacute phase (2 days to weeks after injury), the release of cytotoxic substances and sustained inflammation cause secondary neuronal death and the formation of cystic cavities [4], together with the activation of neuroglia to deposit excess extracellular matrix which contributes to glial scar genesis. Axonal regrowth and remyelination subsequently occur in the chronic phase (weeks to months after injury) [5]. Astrocytes exhibit cellular heterogeneity during this process [6, 7]. Specifically, reactive astrocytes show cellular hypertrophy and upregulation of glial fibrillary acidic protein (GFAP), an intermediate filament III protein almost exclusively expressed in astrocytes [8]. This heterogeneity invoked new explorations of their double-sided functions other than their conventional roles in glial scar formation, which act as physical and biochemical barriers for the migration of neural precursor cells, as well as for axonal regrowth and remyelination [9, 10]. Functionally, reactive astrocytes have been reported to induce axon sprouting and regulate synapse plasticity and circuit reorganization [7], while scar-forming astrocytes show elongated morphology and aid in axon regeneration [7, 11].

Furthermore, cognitive deficits and emotional disorders are common in individuals with SCI [12, 13]. Various experimental models of SCI exhibit impairments similar to those observed clinically [14–19]. Activation of microglia was detected in the hippocampus 12 weeks after spinal cord contusion [15], whereas neuronal ER stress and neurodegeneration were observed in the cortex, hippocampus, and thalamus 16 weeks after spinal cord contusion [16]. On the other hand, reactive astrocytes, increased microglia numbers, and enhanced death of amplifying neural progenitors were observed in the hippocampus 7 days after spinal cord compression [18]. Long-term after spinal cord compression, astrocytes remained reactive, the number of ramified surveillance microglia decreased, the number of hypertrophic arginase-1 negative microglia increased, and the frequencies of dividing neural stem cells and amplifying neural progenitors dropped in the hippocampus [18, 19]. These abnormalities certainly contribute to cognitive deficits and emotional disorders. However, whether other encephalic region(s) get involved remains unknown.

To study the spatial and temporal activation of astrocytes after SCI, we constructed a GFAP-IRES-Venus-AkaLuc mouse model via the knock-in (KI) technique [20, 21]. These mice are applicable to both in vivo AkaLumine bioluminescence imaging and ex vivo microscopy detecting the Venus protein. In our preliminary experiments, however, the augmentation of bioluminescence in mouse brains was detected after complete spinal cord transection (SCT) surgery, which was even earlier and greater in magnitude than that in post-SCT spinal cords. This phenomenon has never been reported to the best of our knowledge. Meanwhile, the bulk-sequencing of brain tissue from the same SCT-treated GFAP-labeled mice revealed the activation of apoptotic signaling and upregulation of serum and glucocorticoid-regulated kinase 1 (SGK1), which is inducible by hormones such as mineralocorticoids and glucocorticoids [22, 23].

The hypothalamic-pituitary-adrenal (HPA) axis is known as one of the canonical stress hormone systems functioning in response to external pressures [24]. Glucocorticoids, as the terminal effectors of the HPA axis, reportedly function in various physiological processes by binding to the bioactive glucocorticoid receptor-alpha (GR-α). GR-α is widely expressed in neurons within the limbic system, and the neuronal GR-α activated by elevated glucocorticoids during neurological disorders has been reported to attenuate neuronal excitability and neuronal survival through various transcriptional regulatory mechanisms, such as the activation of p53-dependent mitochondrial apoptosis [25], negative feedback regulation of brain-derived neurotrophic factor (BDNF) [26], and suppression of neurogenesis by inhibiting PI3K-AKT signaling [27]. Based on the results of our preliminary experiments and the reported findings described above, we aimed to explore the potential mechanisms underlying GFAP and SGK1 upregulation in the brain after SCI, and the potential relationship between SGK1 upregulation and cellular apoptosis.

## Materials and methods

### Animal procedures

**Fig. 1:**
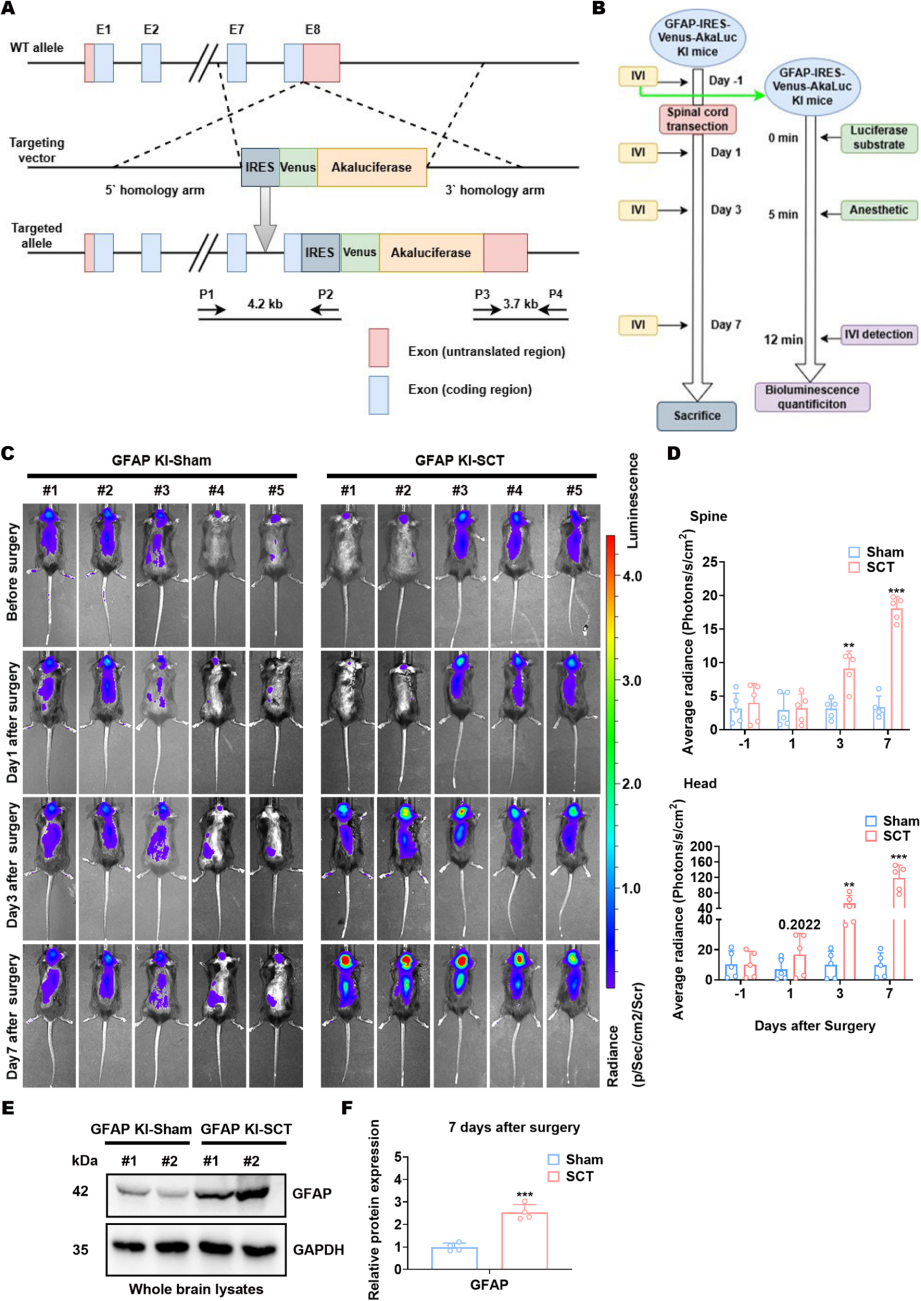
GFAP signals are intensified in mouse brains after spinal cord transection. **A** Schematic representation of the mouse GFAP genomic locus and the designated targeting strategy used to produce and identify GFAP-IRES-Venus-AkaLuc KI mice. **B** Schematic description of spinal cord transection (SCT) and in vivo imaging procedures for KI mice. IVI, in vivo imaging. **C** Representative images of in vivo imaging detecting the bioluminescence of KI mice at various time points of sham surgery or SCT. **D** Statistical analysis of bioluminescence signals in the spine (top) and head (bottom) of the KI mice shown in (**C**). **p < 0.01, ***p < 0.001 vs. Sham; *n* = 5 biologically independent samples. **E**, **F** Protein expression of GFAP in the whole-brain lysates of KI mice 7 days after sham surgery or SCT. Representative images (**E**) and statistical analyses (**F**) are shown. ***p < 0.001 vs. Sham-whole brain; *n* = 4 biologically independent samples.

All animal procedures were designed and performed according to the regulations of the Institutional Animal Care and Use Ethics Committee of the Beijing Institute of Basic Medical Sciences under the ethics approval number AMMS 2021-1356. The establishment of GFAP-IRES-Venus-AkaLuc knock-in (KI) C57BL/6 mice was implemented by Shanghai Model Organisms Center, Inc. (contract ID: N1-2688) which generated F0 founder animals (Fig. 1A). Then, heterozygous and homozygous KI offsprings were bred, identified, and maintained at the Animal Experiment Center of the Beijing Institute of Basic Medical Sciences. The randomization of all tested mice was performed before any treatment. Briefly, the body weights of mice were measured. Then, the mice were numbered by body weights successively and aligned to designated experimental groups by the random number table method. The complete SCT model was constructed according to a previously described method [28]. In brief, 8-week-old C57BL/6 male mice were anesthetized and placed in the prone position. Midline incisions on the back and blunt dissection were performed to expose the thoracic level 8 (T8) - thoracic level 10 (T10) segment of the spinal spinous process. Then, laminectomy was performed at T10 and the spinal cord was completely transected using fine microscissors. Control mice underwent the same procedure except for the transection (sham surgery). The post-surgery mice were kept in separate cages with regular antibiotic administration, artificial urination, and artificial defecation. Each SCT-treated mouse was evaluated by the Basso Mouse Scale for Locomotion (BMS) [29]. Only mice entirely incapable of hindlimb motor function and tactile sensation were sacrificed to obtain the brains 7 days after surgery (Videos S1, 2). Each experimental group included at least four surviving mice for sampling. Frozen sections of the harvested brains were subjected to immunofluorescence staining, and the remaining tissues were subjected to immunoblotting assays. The investigators were not blind to the group allocation during the animal experiments.

### In vivo imaging (IVI)

In vivo imaging of sham- or SCT-treated KI mice was performed with an IVIS ^®^ Spectrum system (PerkinElmer, Santa Clara, CA, USA). The hair of the mouse was shaved off the detection sites to exclude potential interference. 50 μl of PBS solution of luciferase substrate AkaLumine-HCL (TokeOni, Sigma-Aldrich, #808350) at 300 μM was administered to each mouse i.p. before testing. 5 min later, the mice were anesthetized and placed in the detection chamber in the prone position. 12 min after the administration of AkaLumine solution, bioluminescence imaging was performed (parameters are as follows: open for total bioluminescence, exposure time = auto, binning = medium: 8, field of view = 25.2 × 25.2 cm, and f/stop = 1) and the corresponding quantifications were executed by Living Image 4.5.2. Software (PerkinElmer, Santa Clara, CA, USA) for statistics.

### Bulk RNA-sequencing and bioinformatic analysis

7 days after sham surgery or SCT, the KI mice were anesthetized, followed by thoracotomy and intracardiac perfusion with precooled saline. Then, whole brains were isolated and homogenized with TRIzol^®^ reagent (ThermoFisher Scientific, #15596026). The integrity of the extracted total RNA was assessed by a Bioanalyzer 2100 (Agilent, CA, USA) and electrophoresis. Poly(A) RNA was purified from 1 μg total RNA per sample using Dynabeads Oligo (dT) 25 (ThermoFisher Scientific, #61005) and fragmented into small pieces using the Magnesium RNA Fragmentation Module (New England Biolabs, e6150). The cleaved RNA fragments were reverse-transcribed to cDNA by SuperScript™ III Reverse Transcriptase (ThermoFisher Scientific, #1896649), which was subsequently used to synthesize U-labeled second-stranded DNAs with *E. coli* DNA polymerase I (New England Biolabs, m0209), RNase H (New England Biolabs, m0297) and dUTP Solution (ThermoFisher Scientific, R0133). An A-base was then added to the blunt ends, preparing them for ligation to the indexed adapters. Single- or dual-index adapters were ligated to the fragments, and size selection was performed with AMPureXP beads. After heat-labile UDG enzyme (New England Biolabs, m0280) treatment of the U-labeled second-stranded DNAs, the ligated products were amplified with PCR. The average insert size for the final cDNA library was 300 ± 50 bp. Finally, the 2 × 150 bp paired-end sequencing was performed on an Illumina NovaSeq™ 6000 system (LC-Bio Technology Co., Ltd., Hangzhou, China) following the vendor’s recommended protocol. The unprocessed raw data derived from the Illumina NovaSeq™ 6000 system have been uploaded to the Sequence Read Archive (SRA, PRJNA1136156) and released.

Fastp software (https://github.com/OpenGene/fastp) was used to remove the reads that contained adapter contaminants, low-quality bases, and undetermined bases with the default parameters. Then, HISAT2 (https://ccb.jhu.edu/software/hisat2) was used to map reads to the reference genome (*Mus musculus* GRCm38). The mapped reads were subsequently assembled using StringTie tools (https://ccb.jhu.edu/software/stringtie) with default parameters. Then, all the transcriptomes from all samples were merged to reconstruct a comprehensive transcriptome with gffcompare tools (https://github.com/gpertea/gffcompare/). After the final transcriptome was generated, StringTie was used to estimate the expression levels of all transcripts by calculating the FPKM (FPKM = [total exon fragments/mapped reads (millions) × exon length (kB)]). Differentially expressed genes (DEGs) were selected with fold changes >2 or <0.5 and with parametric F-test comparing nested linear models (*p* < 0.05) via the R package edgeR (https://bioconductor.org/packages/release/bioc/html/ edgeR.html). The GO and KEGG enrichment analyses of DEGs were implemented with DAVID tools (https://david.ncifcrf.gov/).

### Adeno-associated virus (AAV) construction and stereotaxic injection

To further investigate the potential role of DEG *Sgk1* identified by bulk RNA-sequencing, AAVs designated to silence SGK1 expression specifically in mature neurons were obtained from Shanghai Genechem Co., Ltd., for stereotaxic injection in the frontal association cortex (FrA). Three distinct short hairpin RNAs (shRNAs) targeting mouse SGK1 (SGK1-sh1: ttcttcaacagaacattccgc, SGK1-sh2: ctcaggagccagatactcagg, and SGK1-sh3: atccttggcacccagcctctt) were separately enveloped into the virus vector rAAV2/9-hSyn promoter-eGFP-MIR155(MCS)-WPRE-SV40 PolyA, and the sequence with the highest knockdown efficiency according to in vitro infection was chosen for subsequent stereotaxic injection. AAV injection into KI mice was performed three weeks prior to SCT surgery. The injection site was set based on the stereotaxic coordinates from the Mouse Brain Atlas (mediolateral/ML = 1.5 mm, anteroposterior/AP = ± 3 mm, dorsoventral/ DV = −2 mm).

### Cell culture, transfection, and transduction

To verify various anti-GR antibodies and to evaluate the knockdown efficiency of AAV-based murine SGK1 shRNAs, human embryonic kidney 293T cells and murine neuronal HT22 cells were employed for in vitro transfection and transduction, respectively. 293T cells and HT22 cells (Cell Resource Center, Institute of Basic Medical Sciences, CAMS & PUMC) were maintained in complete DMEM (10% FBS, 1% penicillin-streptomycin) and incubated under standard conditions (37 °C and 5% CO_2_). Hoechst staining was performed quarterly for the detection of mycoplasma contamination, and no extra-nuclear staining was found. HT22 cells were transduced with AAV carrying nontargeting control (NC) shRNA (AAV-NC), AAV-SGK1-sh1, AAV-SGK1-sh2, or AAV-SGK1-sh3 at an optimized multiplicity of infection (MOI) of 5 × 10^6^. 293T cells were transfected with pcDNA3.1, pcDNA3.1-Myc-mouse GR-α (NM_001361209.1), or pcDNA3.1-Myc-mouse GR-β (HM236293.1) with Lipofectamine^TM^ 2000 reagent (ThermoFisher Scientific, #11668019). Transduced or transfected cells were subjected to subsequent detections 48 h later.

### Compounds

The GR antagonist mifepristone (Selleck, S2606) was used for in vivo study, according to previous reports [30, 31]. Briefly, mice were administered with mifepristone (10 mg/kg) i.p. one day before SCT. The same treatment was repeated every two days until the mice were sacrificed to maintain the inhibitory effects. Then, FrA tissues were isolated and subjected to immunoblotting. What’s more, dexamethasone (Solarbio, ID0170) treatment (1 μM, 36 h) of HT22 cells was employed to mimic the in vivo effects of glucocorticoid stimulation, as this procedure has been reported to induce the apoptosis of HT22 cells [32, 33].

### Quantitative real-time PCR (qPCR)

Total RNA samples used for bulk RNA-sequencing were also applied to quantitative real-time PCR. Reverse transcription was performed with a PrimeScript^TM^ RT reagent kit (Takara #RR036A) on a Bio-Rad T100 thermal cycler, and the qPCR procedure was performed with Taq Pro Universal SYBR qPCR Master Mix (Vazyme, Q712-02), all according to the manufacturer’s instructions. The primer sequences (synthesized by Sangon Biotech Inc., China) are shown in Table S1. qPCR assays were conducted on an Applied Biosystem StepOne instrument. The mRNA levels of target genes were quantified by the 2^-ΔΔCt^ method, with *Gapdh* used as the housekeeping gene for normalization.

### Immunofluorescence (IF)

Cell samples seeded in glass bottom Petri dishes were fixed with 4% paraformaldehyde (PFA) for 10 min. For tissue sectioning, mice were anesthetized, followed by thoracotomy and successive intracardiac perfusion with precooled saline and 2% PFA. Then, the whole brains were isolated, fixed with 4% PFA for an additional 24 h, and dehydrated with a graded series of sucrose solutions. After that, brain samples were embedded and sliced with a Dakewe CT520 cryostat microtome with a section thickness of 8 μm. Coronal sections of the prefrontal cortex, diencephalon, and hippocampus were stored at −80 °C before staining. Fixed cell samples or tissue sections were treated with 0.3% Triton X-100 in PBS for 10 min, blocked with donkey serum (Solarbio #SL050) for 30 min, incubated with primary antibodies O/N at 4 °C and then with fluorescence-conjugated secondary antibodies at RT for 2 h. Afterward, 4′,6-diamidino-2-phenylindole (DAPI, FluoroPure™ grade, Invitrogen, D21490) was applied to stain the nuclei, and the samples were mounted onto glass slides and imaged by an Olympus IX53 fluorescence microscope, a Leica SP8 confocal microscope, an Oxford Instruments Dragonfly 400 confocal microscope or an Olympus VS200 slide scanner. The antibodies used are listed in Table S2.

### Immunoblotting (IB)

Harvested brain tissues were homogenized in RIPA buffer (50 mM Tris-Cl, pH 7.5, 1% NP40, 0.35% deoxycholate, 150 mM NaCl, 1 mM EDTA, 1 mM EGTA, supplemented with the protease and phosphatase inhibitor cocktail). Lysates were probe sonicated and centrifuged (12,000 × *g* at 4 °C for 15 min). A Pierce^®^ BCA assay (ThermoFisher Scientific #23225) was used to determine the protein concentrations of the lysates and 30 μg of each sample was loaded into SDS-PAGE gels and electrophoresed. The separated proteins were blotted onto a 0.45 μm PVDF membrane (Amersham^TM^, #10600023) and blocked with 5% w/v dry skim milk in TBS solution for 2 h. Primary antibody incubations were then performed with antibody working solutions in 3% w/v BSA in TBST at 4 °C O/N, followed by incubation with secondary antibodies in TBST antibody working solutions at RT for 2 h. The antibodies used are listed in Table S2. Nuclear protein samples were separated from whole-cell lysates using an NE-PER™ kit. GAPDH and Lamin B were selected as IB loading controls for the cytoplasmic and nuclear extracts, respectively. The membrane exposure was performed by ECL (ThermoFisher Scientific, #34580 or #34095), and a ClinX 6200 touch imager, while the quantifications were performed with ImageJ software.

### Apoptosis detection

Neuronal apoptosis in the prefrontal sections of SCT-treated mice was verified by the co-staining with a TdT-mediated dUTP nick-end labeling (TUNEL) kit (Beyotime, C1090) and an anti-cleaved-Caspase-3 antibody (R&D, AF835), according to the instructions of the manufacturers. Then, the samples were stained with DAPI, mounted, and imaged by an Olympus IX53 fluorescence microscope or an Olympus VS200 slide scanner. The TUNEL^+^cleaved-Caspase-3^+^/DAPI^+^ ratios in three random fields of each prefrontal section were counted for statistics. For the cellular apoptosis of cultured HT22 cells, both TUNEL and JC-1 staining (Beyotime, C2003) were applied for the evaluation. Live HT22 cells stained with JC-1 probes were imaged by an Olympus IX53 fluorescence microscope.

### Enzyme-linked immunosorbent assay (ELISA)

The serum levels of cortisol in C57 mice that received SCT surgery were measured by a competitive ELISA kit (Cayman, #500360) [34], according to the instructions of the manufacturer. The absorbances at 405 nm were read by a VICTOR^TM^ X3 plate reader (PerkinElmer, USA), and the cortisol concentrations in the test samples were calculated according to the standard curves generated by 4-parameter logistic curve fitting with Origin 9.1 software.

### Tissue clarity and light sheet microscopy

7 days after sham surgery or SCT, the whole brains of the KI mice were harvested after thoracotomy and intracardiac perfusion. The harvested brain samples were fixed with 4% PFA with shaking at 4 °C for 24 h, washed in PBS for 1 h twice, then subjected to a reported tissue-clearing protocol compatible with immunostaining named iDISCO (Fig. S1) [35] or a reported passive immersion tissue-clearing protocol named PEGASOS [36]. Briefly, samples were applied to the pretreatments without methanol, immunolabeling, and tissue clearing successively and stored in dibenzyl ether (DBE) (Sigma 108014-1KG) at RT. The stained and tissue-cleared samples were imaged with a Zeiss Lattice Lightsheet 7 microscope equipped with two-sided 5×/0.1 NA illumination optics and a 5×/0.16 NA detection optic. The 3D images of whole brains reconstructed with the Zeiss ZEN blue software were reformatted with ImarisViewer 10.0.0 software for representation (Video S3).

### Behavioral tests

The depressive-like behaviors and hindlimb locomotion of post-SCT mice were evaluated via sucrose preference tests (SPTs), BMS scoring, and gait analysis, respectively. For the assessment of hindlimb locomotion, sham/SCT-treated mice preinjected with AAV-NC or AAV-shSGK1 vectors were subjected to successive BMS scoring from open-field tests weekly after surgery [28]. On day 28 after surgery, the gaits of the mice were also recorded and applied to a commercialized gait analysis and processing system (ZS-BT/S, Beijing Zhongshidichuang Technology and Development), on which parameters describing hindlimb locomotion were quantified and used for statistics [37]. As for the measurement of depressive-like behaviors, the SPT was performed 1 day before surgery and 7 days after surgery, respectively, according to a published protocol [38].

### Statistical analysis

All data were collected from at least three biological replicates. Quantitative data were presented as the mean ± SEM and analyzed using GraphPad Prism software V8.0. Two-tailed Student’s *t*-tests or one-way ANOVA (Tukey’s test) were used to evaluate the quantitative variables that passed the normality test (Shapiro–Wilk test) and homogeneity of variance test (Bartlett’s test). Wilcoxon rank-sum test was used to evaluate the quantitative variables that failed to pass the Shapiro–Wilk test. Brown-Forsythe and Welch ANOVA were used to evaluate the quantitative variables that failed to pass Bartlett’s test. *p*-values < 0.05 were considered statistically significant.

## Results

### GFAP signal is intensified in the mouse brain after spinal cord transection

To study the spatial and temporal activation of astrocytes after SCT, we constructed a GFAP-IRES-Venus-AkaLuc KI mouse model (Fig. 1A). Compared to conventional luciferase, Akaluc bioluminescence provides superior sensitivity for tracking in vivo dynamics within the CNS [39]. The constructed KI mice were subjected to complete SCT, and in vivo, imaging was performed at time points designated for the acute and subacute phases after SCT (Fig. 1B). As expected, SCT, but not sham surgery, led to elevated bioluminescence intensities in the mouse spinal cord region on days 3 and 7 after surgery (Fig. 1C, D). Surprisingly, SCT-treated mice showed much more remarkable augmentation of the bioluminescence intensities in the head region than in the spinal cord region on days 3 and 7 after surgery (Fig. 1C, D). Furthermore, the bioluminescence intensities in the head region of SCT-treated mice tended to be greater than sham-treated mice even on day 1 after surgery (Fig. 1C, D). Immunoblotting analysis of whole-brain lysates confirmed the elevated protein levels of GFAP 7 days after SCT (Fig. 1E, F). This GFAP upregulation in the CNS might suggest neuroinflammation triggered by post-SCT disease conditions, which requires further investigation.

### SGK1 upregulation and apoptotic signaling are activated in the mouse brain after spinal cord transection

**Fig. 2:**
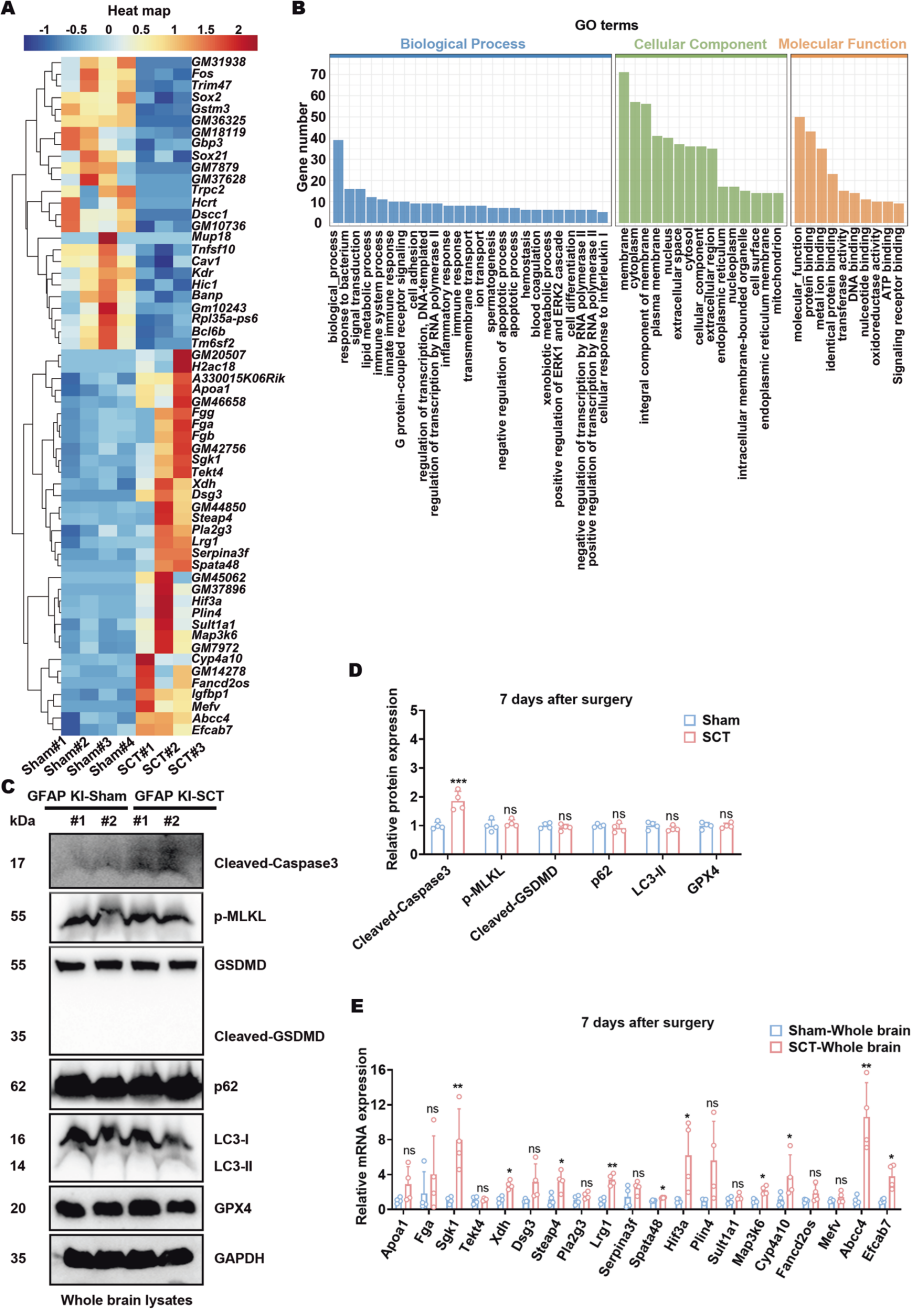
SGK1 upregulation and apoptotic signaling are activated in the mouse brain after spinal cord transection. **A** Heatmap of DEGs generated from bulk RNA-sequencing of whole brains of KI mice 7 days after sham surgery or SCT. **B** Bar plots of the GO enrichment of DEGs shown in (**A**). **C**, **D** Protein expression of cleaved-Caspase-3, p-MLKL (Ser345), GSDMD, p62, LC3, and GPX4 in the whole-brain lysates of KI mice 7 days after surgery. Representative images (**C**) and statistical analyses (**D**) are shown. ***p < 0.001 vs. Sham-whole brain; *n* = 4 biologically independent samples. **E** qPCR analysis of the upregulated genes shown in (**A**). *p < 0.05 and **p < 0.01 vs. Sham-whole brain; *n* = 4 biologically independent samples.

To explore the underlying mechanisms, whole brains of KI mice were harvested 7 days after sham surgery or SCT and subjected to bulk RNA-sequencing. The whole-brain DEGs between sham- and SCT-treated mice were mainly related to stress reactions, signal transduction, neurological disease, neuronal damage, lipid metabolism, etc. (Fig. 2A). In line with this, both apoptotic signaling and apoptotic resistance were identified from the GO enrichment of DEGs (Fig. 2B). Therefore, whole-brain lysates were screened for various biomarkers of programmed cell death. While the levels of p-MLKL, cleaved-GSDMD, p62, LC3-II, and GPX4 in the brains of sham- and SCT-treated mice remained comparable, cleaved-Caspase-3 synchronized with GFAP upregulation (Figs. 2C and 1D), which was also in agreement with the GO enrichment results. Then, qPCR was employed to check the upregulated DEGs, among which *Sgk1* ranked as one of the most stable DEGs (Fig. 2E). *Sgk1* has been reported to be activated as the downstream signaling of mTOR complex 2 [40, 41], which was also in accordance with the mTOR signaling pathways generated from KEGG enrichment of DEGs (Fig. S2). The mRNA level of *Gfap* in the whole brain, however, was not significantly different between sham- and SCT-treated wild-type mice, according to both bulk RNA-sequencing and qPCR (Fig. 2A, Fig. S3). These data suggest that *Gfap* transcription could be region-specific after SCT (upregulated in certain encephalic regions but downregulated in some other encephalic regions).

### GFAP-positive neurons emerge in the mouse prefrontal cortex after spinal cord transection

**Fig. 3:**
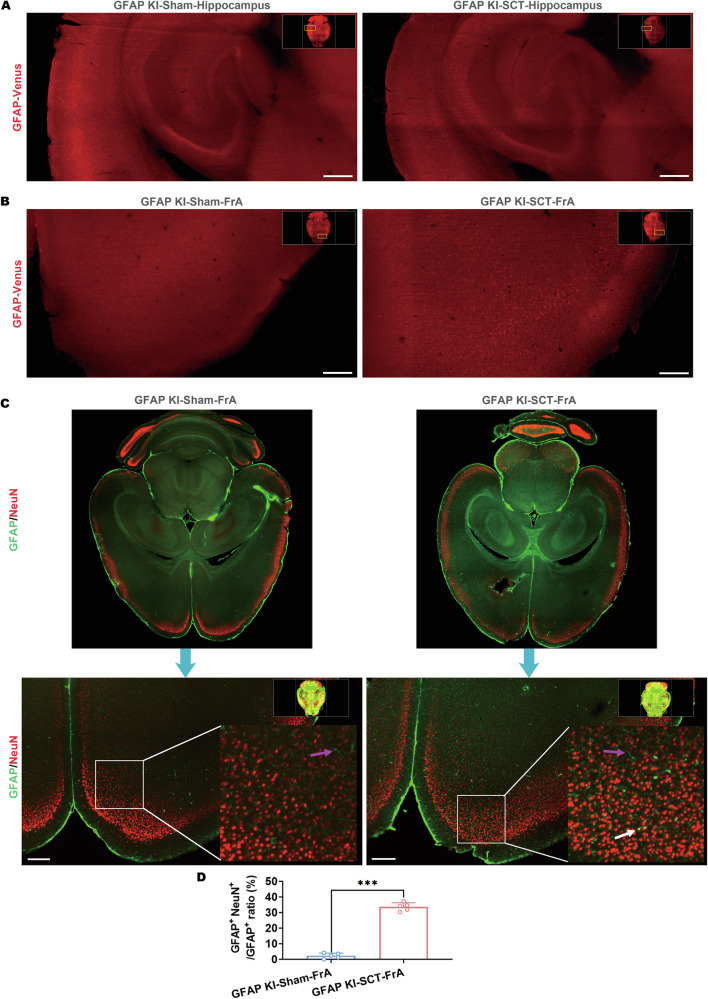
GFAP-positive neurons emerge in the mouse frontal association cortex after spinal cord transection. **A**, **B** Representative horizontal section images of the hippocampus (**A**) and frontal association cortex (FrA, **B**) of an SCT-treated KI mouse and its sham surgery control after tissue clearing without immunostaining (PEGASOS). Scale bar, 500 μm. **C** Representative horizontal section images of the whole brain of an SCT-treated KI mouse and its sham surgery control after immunostaining-compatible tissue clearing (iDISCO) with antibodies against GFAP (green) and NeuN (red). GFAP^+^ NeuN^-^ cells were indicated with magenta arrows whereas GFAP^+^ NeuN^+^ cells were indicated with white arrows. Scale bar, 500 μm. **D** Statistical analysis of GFAP^+^ NeuN^+^ cell ratios in all GFAP^+^ cells within the FrA sections of sham/SCT-treated KI mice. ***p < 0.001 vs. GFAP KI-Sham-FrA; *n* = 5 distinct transverse sections.

In order to further specify the encephalic region(s) with GFAP upregulation after SCT, tissue clearing was applied to KI mice, which is also applicable to ex vivo microscopy detecting the Venus protein. Like other organic-solvent-based tissue-clearing methods, PEGASOS severely mitigated endogenous fluorescent signals [42]. Therefore, the downregulation of Venus could not be observed through light sheet microscopy of the whole brain after PEGASOS procedures. Our results showed no detectable augmentation of Venus or GFAP signals in the hippocampus and basolateral amygdala (Fig. 3A, Fig. S4), in which post-SCT astrogliosis has been reported [43]. The enhancement of Venus signals was observed in the frontal association cortex (FrA) of SCT-treated KI mice (Fig. 3B), an encephalic region located in the prefrontal cortex that is involved in associative learning and recognition memory processes [44, 45]. Moreover, the brains of SCT-treated mice were divided into different encephalic regions for immunoblotting. The results confirmed the GFAP upregulation in the prefrontal cortex of SCT-treated mice observed by light sheet microscopy (Fig. S5). Intriguingly, the morphology of most Venus-expressing cells in the FrA of the SCT-treated KI mice resembled that of neurons, but not astrocytes (Fig. 3B). To confirm this possibility, iDISCO procedures compatible with immunostaining were employed. This method eliminates endogenous fluorescence [35]. The whole-brain tissues were stained with antibodies against GFAP and NeuN. The light sheet microscopy of the whole brain of an SCT-treated mouse revealed that GFAP signals were indeed intensified in the FrA 7 days after surgery (Fig. 3C). Numerous GFAP^+^ cells in this region were also NeuN^+^ (Fig. 3C). Similar GFAP^+^ NeuN^+^ cells were discovered in primary visual cortex (V1) of the cerebrum, as well as areas around the central nucleus of the inferior colliculus (CIC) and dorsal paragigantocellular nucleus (DpG) of the brainstem (Fig. S6). Taken together, these data prompted us to focus on the status of FrA neurons after SCT.

### Cellular apoptosis correlates with SGK1 upregulation in GFAP-positive neurons in the frontal association cortex of mice subjected to spinal cord transection

**Fig. 4:**
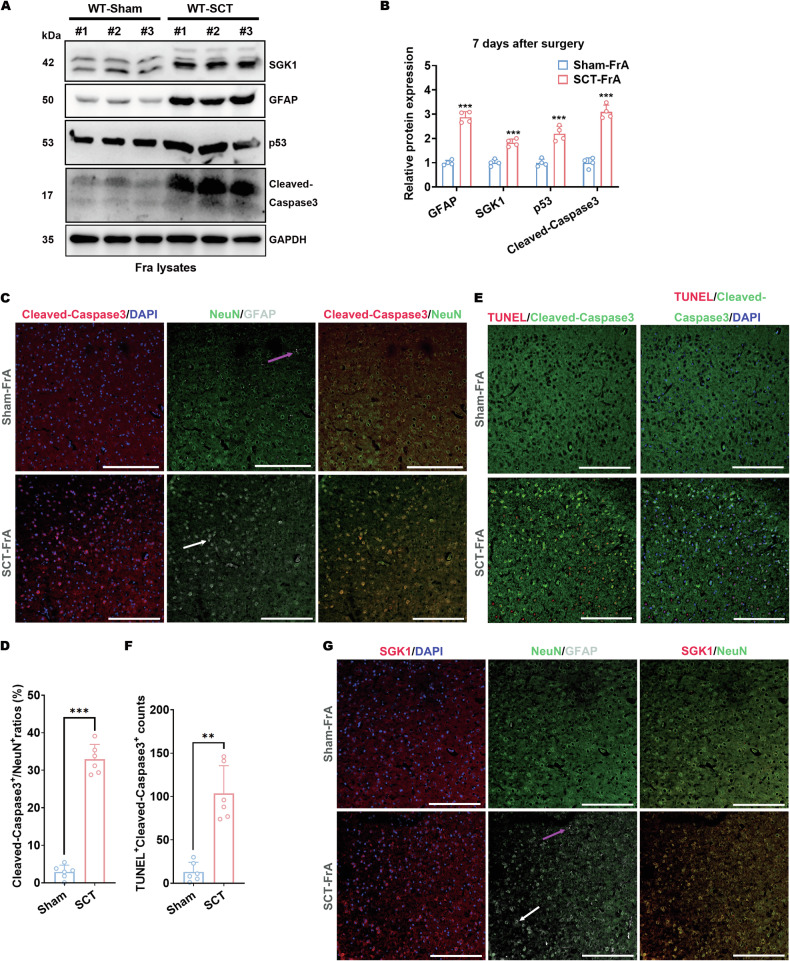
Cellular apoptosis correlates with SGK1 upregulation in GFAP-positive neurons in the frontal association cortex of mice with spinal cord transection. **A**, **B** Protein expression of SGK1, GFAP, p53, and cleaved-caspase-3 in the frontal association cortex tissue from SCT-treated, C57 wild-type mice 7 days after the surgery. Representative images (**A**) and statistical analyses (**B**) are shown. ***p < 0.001 vs. Sham-FrA; *n* = 4 biologically independent samples. Coronal sections of the FrA tissue from sham- or SCT-treated mice 7 days after surgery were subjected to the following co-staining: **C**, **D** Co-staining with antibodies against NeuN (green), GFAP (white) and cleaved-Caspase-3 (red). Representative images (**C**, scale bar, 250 μm) and statistical analyses (**D**) are shown. ***p < 0.001 vs. Sham-FrA; *n* = 6 biologically independent samples. **E**, **F** Co-staining with an antibody against cleaved-Caspase-3 (green) and TUNEL probes (red). Representative images (**E**, scale bar, 250 μm) and statistical analyses (**F**) are shown. **p < 0.01 vs. Sham-FrA; *n* = 6 biologically independent samples. **G** Co-staining with antibodies against NeuN (green), GFAP (white), and SGK1 (red). Representative images (scale bar, 250 μm) are shown.

GFAP expression is occasionally observed in neurons undergoing degeneration or damage [46]. Moreover, multiple studies have shown that SGK1 is activated by cellular stress, which negatively regulates neuronal apoptosis in multiple studies [47, 48]. Within the prefrontal cortex, cellular apoptosis occurred mainly in the FrA (Fig. S7), as indicated by TUNEL staining. Thus, the emergence of GFAP^+^ neurons in the FrA might suggest ongoing SGK1-regulated neuronal damage within this encephalic region after SCT. In this scenario, the FrA tissues of SCT-treated wild-type mice were applied to immunoblotting. The protein levels of p53, cleaved-Caspase-3, GFAP, and SGK1 were observed to be synchronously upregulated after SCT (Fig. 4A, B). Additionally, in situ detection of FrA neurons was implemented by immunostaining of brain sections. Co-staining of coronal sections of the prefrontal cortex revealed that most FrA NeuN^+^ cells changed into cleaved-Caspase-3^+^ 7 days after SCT (Fig. 4C, D). These cleaved-Caspase-3^+^ cells were also TUNEL^+^ (Fig. 4E, F), confirming the occurrence of cellular apoptosis. On the other hand, almost all cleaved-Caspase-3^+^ cells in the FrA of SCT-treated mice were NeuN^+^ mature neurons (Fig. 4D). SCT led to significantly increased TUNEL^+^/cleaved-Caspase-3^+^ neuronal cell counts in the FrA than did sham surgery (Fig. 4F). That is, mature neurons, but not glial cells in the FrA are sensitive to SCI. Moreover, the majority of FrA GFAP^+^ cells became SGK1^+^ after SCT (Fig. 4G), and these SGK1^+^ cells after SCT were also NeuN^+^ (Fig. 4G). Thus, we concluded that the ongoing apoptosis of FrA GFAP^+^ neurons after SCT synchronized with SGK1 upregulation, which might activate apoptosis resistance.

### Hypercortisolism after spinal cord transection leads to neuronal apoptosis and the upregulation of GFAP and SGK1

**Fig. 5:**
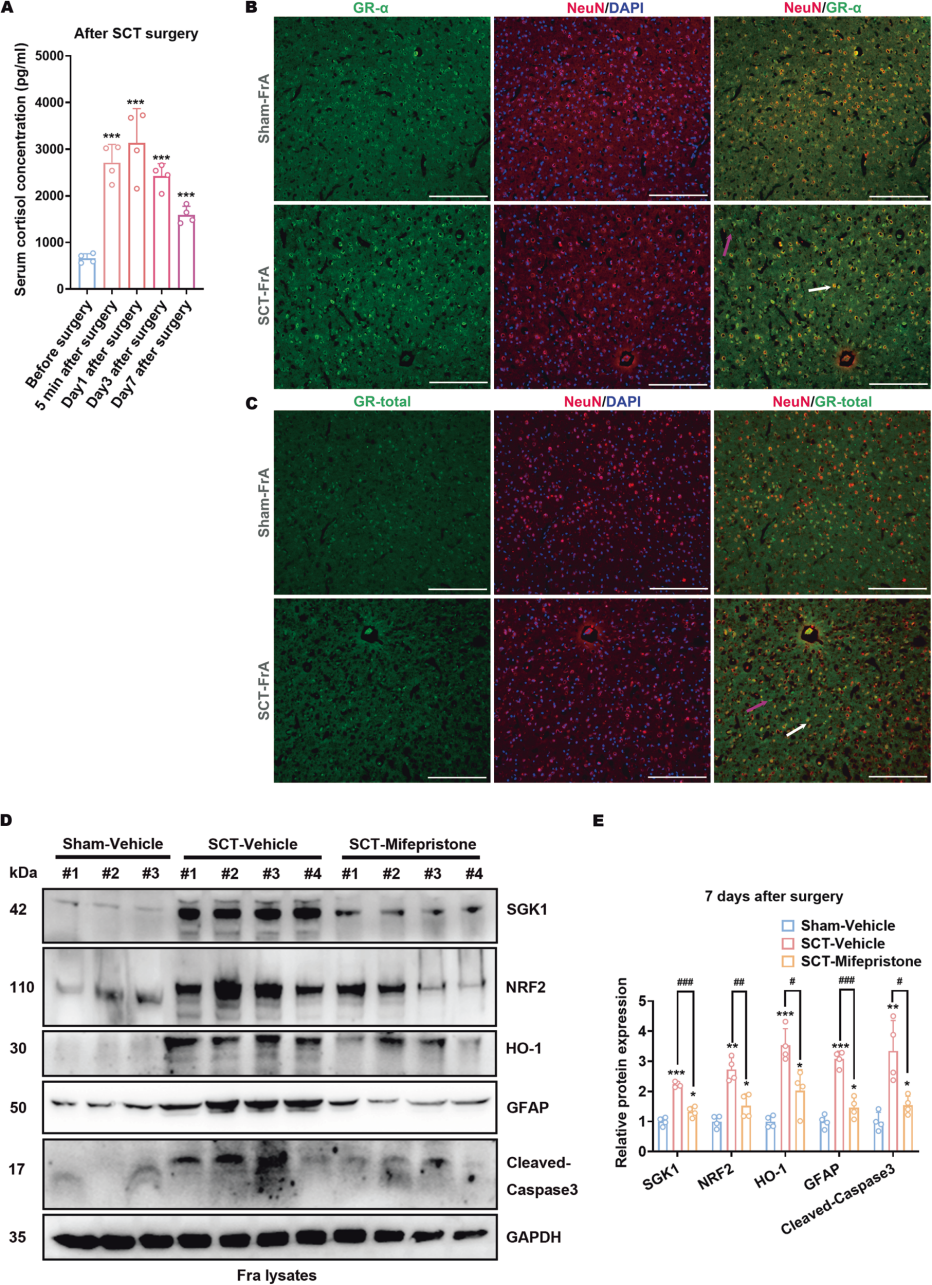
Hypercortisolism after spinal cord transection leads to neuronal apoptosis and the upregulation of GFAP and SGK1. **A** Mouse serum cortisol levels at designated time points before and after SCT surgery were measured by ELISA ***p < 0.001 vs. Sham; *n* = 4 biologically independent samples. **B**, **C** Coronal sections of the FrA tissue from sham- or SCT-treated mice 7 days after surgery were co-stained with antibodies against GR-α (green) and NeuN (red) (**B**) or with antibodies against GR-total (green) and NeuN (red) (**C**). Scale bar, 250 μm. Typical GR-α^+^NeuN^-^/GR-total^+^NeuN^-^ cells were indicated with magenta arrows, whereas GR-α^+^NeuN^+^/GR-total^+^NeuN^+^ cells were indicated with white arrows. **D**, **E** Protein expression of SGK1, NRF2, HO-1, GFAP, and cleaved-Caspase-3 in the FrA of mifepristone-pretreated mice after surgery. Representative images (**D**) and statistical analyses (**E**) are shown. *p < 0.05, **p < 0.01 and ***p < 0.001 vs. Sham-Vehicle; ^##^*p* < 0.01 and ^###^*p* < 0.001 vs. SCT-Vehicle; *n* = 4 biologically independent samples.

Hypercortisolism has long been considered the result of stress reactions and immune dysregulation after SCT [49, 50]. Since either cortisol penetration into the CNS or apoptotic stress could independently trigger SGK1 expression, it is vital to specify the incentive factor(s) for SGK1 upregulation in FrA GFAP^+^ neurons after SCT. According to the ELISA results, the serum level of cortisol dramatically increased 5 min after the transection of the spinal cord peaked on day 1 after surgery, and remained higher than that before the transection until day 7 after surgery (Fig. 5A). Meanwhile, the expression of GR-α in the FrA was detected mainly in GFAP^+^ neurons (Fig. 5B) that underwent apoptosis as demonstrated above. Because the expression of GR was not suppressed by hypercortisolism after SCT (Fig. 5B, C; Fig. S8), most likely no glucocorticoid resistance occurred during this process. In this scenario, the GR antagonist mifepristone was introduced to SCT-treated mice to clarify the effects of hypercortisolism. Immunoblotting showed that the upregulation of cleaved-Caspase-3, GFAP, and SGK1 in the FrA of SCT-treated mice was partially reversed by repeated mifepristone administration (Fig. 5D, E). Thus, hypercortisolism after SCT leads to neuronal apoptosis as well as the upregulation of GFAP and SGK1. Additionally, SCT treatment also enhanced the levels of NRF2, and HO-1 (Fig. 5D, E), downstream anti-apoptotic effectors of SGK1 [51]. The augmentation of these anti-apoptotic effectors after SCT was also mitigated by mifepristone treatment (Fig. 5D, E). Thus, cortisol-induced SGK1 upregulation in GFAP^+^ neurons might partially antagonize cortisol-induced apoptosis.

### SGK1 upregulation exerts neuroprotective effects against glucocorticoid-mediated neuronal apoptosis after spinal cord transection

**Fig. 6:**
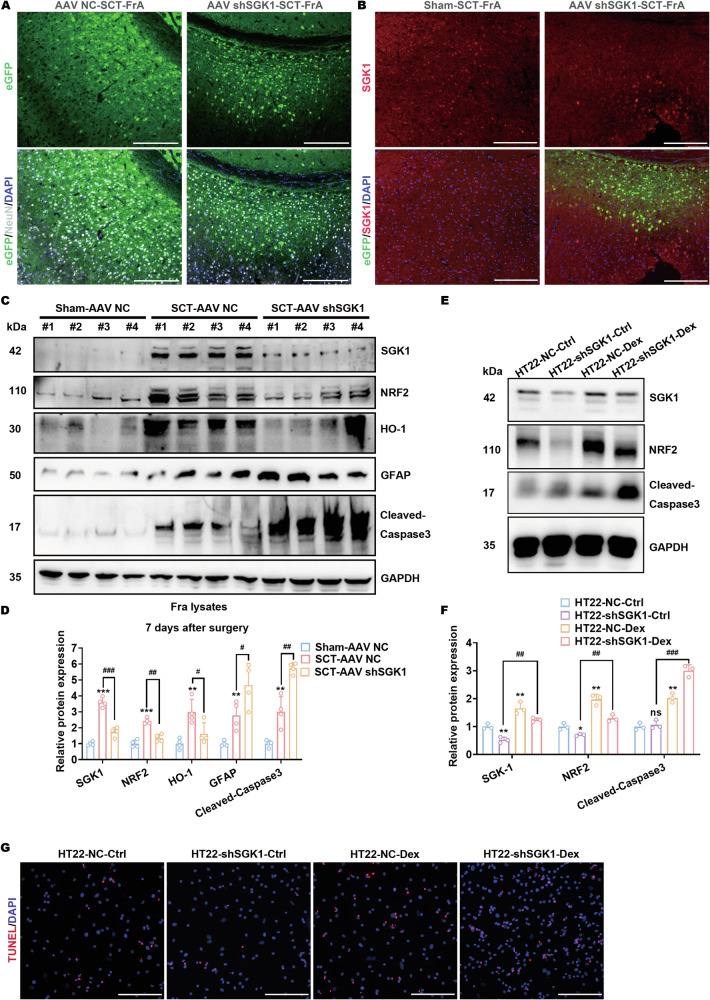
SGK1 upregulation exerts neuroprotective effects against glucocorticoid-mediated neuronal apoptosis after spinal cord transection. **A** Coronal sections of the FrA from SCT-treated mice preinjected with AAV-NC or AAV-shSGK1 carrying the eGFP encoding sequence were stained with anti-NeuN (white). Scale bar, 250 μm. **B** Coronal sections of the FrA from SCT-treated mice preinjected with AAV-shSGK1 were stained with anti-SGK1 (red). Scale bar, 250 μm. **C**, **D** Protein expression of SGK1, NRF2, HO-1, GFAP, and cleaved-Caspase-3 in the FrA of SCT-treated mice preinjected with AAV-NC or AAV-shSGK1. Representative images (**C**) and statistical analyses (**D**) are shown. **p < 0.01 and ***p < 0.001 vs. Sham-AAV NC; ^##^*p* < 0.01, ^###^*p* < 0.001 vs^.^ SCT-AAV NC; *n* = 4 biologically independent samples. (E and F) Protein expression of SGK1, NRF2, and cleaved-Caspase-3 in AAV-shSGK1-infected HT22 cells treated with dexamethasone. Representative images (**E**) and statistical analyses (**F**) are shown. *p < 0.05, **p < 0.01 and ***p < 0.001 vs. HT22-NC-Ctrl; ^##^*p* < 0.01, ^###^*p* < 0.001 vs. HT22-NC-Dex; *n* = 3 biologically independent samples. **G** TUNEL staining of AAV-shSGK1-infected HT22 cells treated with dexamethasone. Scale bar, 500 μm.

To further verify the protective roles of SGK1 upregulation in GFAP^+^ neurons against SCT-induced neuronal apoptosis, we stereotaxically injected AAVs into the FrA to manipulate SGK1 expression. The effectiveness of these AAV vectors in knocking down SGK1 was tested both in vitro and in vivo (Figs. S9 and S10). At the injection site in the FrA, AAV-shSGK1 with the human Syn promoter was proved to specifically infect neurons and silence the neuronal expression of SGK1 after SCT surgery (Fig. 6A, B; Fig. S11). In comparison, infection with AAV-NC without SCT surgery did not contribute to any neuronal apoptosis (Fig. S12). Then, lysates from the FrA of SCT-treated mice preinjected with AAV-shSGK1 were subjected to immunoblotting. Similar to the results shown in Fig. 5D, E, SCT surgery led to elevated levels of SGK1, cleaved-Caspase-3, GFAP, NRF2, and HO-1 in mice preinjected with AAV-NC, compared to those in the sham group (Fig. 6C, D). The injection of AAV-shSGK1 and successful SGK1 silencing in the FrA, however, partially reversed the increases in NRF2 and HO-1 levels and further augmented GFAP and cleaved-Caspase-3 (Fig. 6C, D). These findings suggested that SGK1 was responsible for the activation of NRF2 signaling, which is presumed to restrain the apoptosis of SGK1-upregulated, GFAP^+^ neurons. Then, we tested this hypothesis in vitro, using dexamethasone to mimic the effects of cortisol, which is supposed to initiate neuronal injury. In HT22 cells, dexamethasone treatment was proved to promote the expression of SGK1, cleaved-Caspase-3, and NRF2 (Fig. 6E, F), decreased mitochondrial membrane integrity and preceded apoptosis, as indicated by TUNEL and JC-1 staining, respectively (Fig. 6G and Fig. S13). These dexamethasone-induced cleaved-Caspase-3 expressions decreased mitochondrial membrane integrity and preceded apoptosis were all aggravated by the infection with AAV-shSGK1 (Fig. 6D−G; Fig. S13).

### SGK1 knockdown in mouse FrA neurons aggravated the post-SCI depressive-like behaviors

**Fig. 7:**
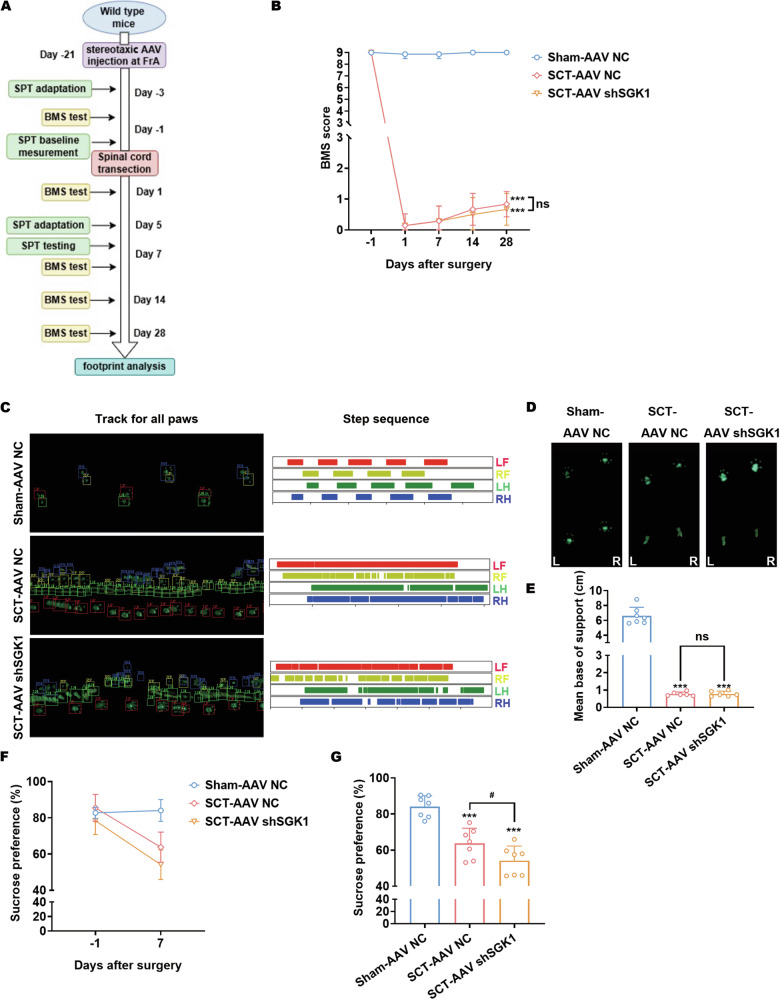
SGK1 knockdown in mouse FrA neurons aggravated the post-SCI depressive-like behaviors. **A** Schematic representation of functional and behavioral tests evaluating SCT-treated mice preinjected with AAV-NC or AAV-shSGK1 in the FrA. **B** BMS scores from open-field tests evaluating hindlimb locomotion of sham/SCT-treated mice preinjected with AAV-NC or AAV-shSGK1. The tests were performed weekly after surgery. ***p < 0.001 vs. Sham-AAV NC; *n* = 6–7 biologically independent samples (One mouse in the SCT-AAV NC group and one mouse in the SCT-AAV-shSGK1 group died during day 14–day 28 after surgery). Wilcoxon rank-sum test was used for statistics. **C** Representative images of gait analysis evaluating hindlimb locomotion of sham/SCT-treated mice preinjected with AAV-NC or AAV-shSGK1, performed 4 weeks after surgery (LF, left front; LH, left hind; RF, right front; RH, right hind). **D**, **E** Footprints and the corresponding average stride length of sham/SCT-treated mice preinjected with AAV-NC or AAV-shSGK1, obtained from the gait analysis. Representative images (**D**) and statistical analyses (**E**) are shown. ***p < 0.001 vs. Sham-AAV NC; *n* = 6–7 biologically independent samples as stated above. Brown-Forsythe and Welch ANOVA was used for statistics. **F**, **G** Sucrose preference tests of sham/SCT-treated mice preinjected with AAV-NC or AAV-shSGK1. The sucrose preference before and after surgery was demonstrated (**F**) and the effects of SGK1 knockdown were analyzed. ***p < 0.001 vs. Sham-AAV NC; ^#^*p* < 0.05 vs. SCT-AAV NC; *n* = 7 biologically independent samples.

Since that SGK1 intervention has been observed to protect GFAP^+^ apoptotic FrA neurons from post-SCI apoptosis, we would like to further investigate its potential effects on the functional and emotional disorders of SCT-treated mice (Fig. 7A). As for the evaluation of locomotive functions, the Basso Mouse Scale (BMS) was applied weekly. As expected, SCT led to a complete loss of hindlimb motor function (Fig. 7B). Then, hindlimb motor function gradually recovered weakly (Fig. 7B). However, there was no difference in hindlimb locomotion between SCT-AAV NC mice and SCT-AAV-shSGK1 mice within 4 weeks after surgery (Fig. 7B). To confirm this, gait analysis was applied 4 weeks after surgery, which also revealed no no difference in hindlimb locomotion between SCT-AAV NC mice and SCT-AAV-shSGK1 mice (Fig. 7C−E). For the behavioral evaluations, we chose the sucrose preference test, due to the fact that the limited hindlimb locomotion of SCT-treated mice made several behavioral experiments unable to be performed, including the Morris water maze, the open-field test, the elevated plus maze, the Porsolt forced swimming test, etc. As expected, SCT resulted in the loss of sucrose preference (Fig. 7F). Notably, SCT-AAV-shSGK1 mice exhibited significantly diminished sucrose preference compared with SCT-AAV NC mice (Fig. 7F, G), which indicated that the SGK1 intervention in FrA neurons might influence the progression of post-SCI anhedonia [43]. This discovery is consistent with the current viewpoints that FrA participates in the consolidation of auditory fear conditioning [52] and associative memory [44].

### SGK1 signaling causes NRF2 intranuclear translocation and HO-1 upregulation in apoptotic neurons

**Fig. 8:**
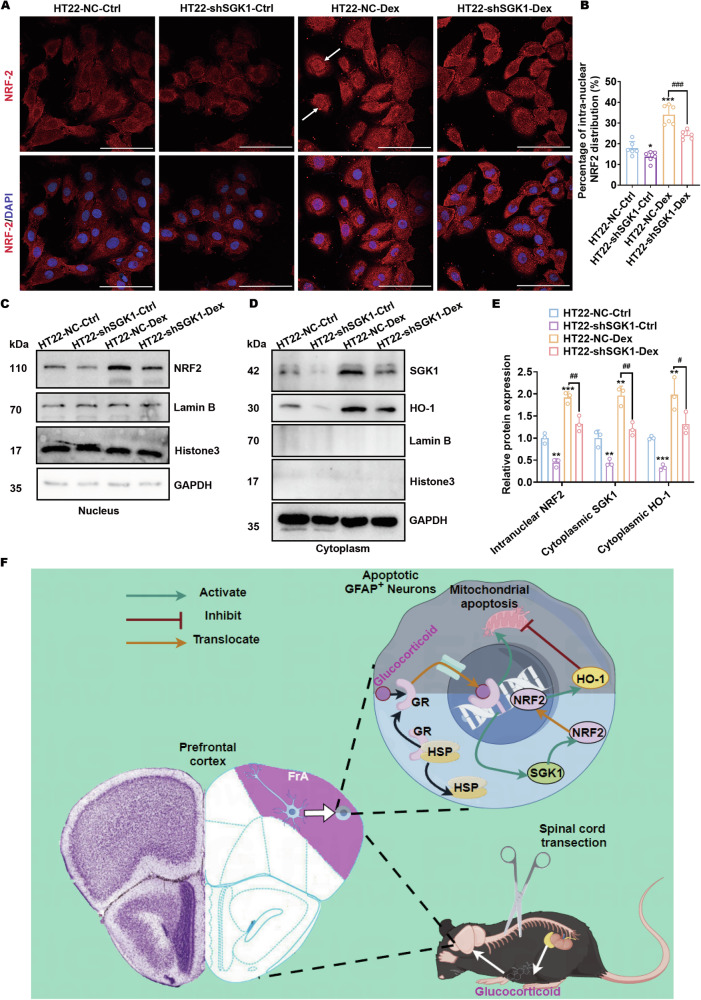
SGK1 signaling causes NRF2 intranuclear translocation and HO-1 upregulation in apoptotic neurons. **A**, **B** AAV-NC- or AAV-shSGK1-infected HT22 cells treated with or without dexamethasone were stained with an antibody against NRF2 (red). Representative confocal microscopy images (**A**, scale bar, 100 μm) and the corresponding analysis of intranuclear NRF2 distribution (**B**) are shown. *p < 0.05 and ***p < 0.001 vs. HT22-NC-Ctrl; ^###^*p* < 0.001 vs. HT22-NC-Dex; *n* = 6 biologically independent samples. Typical nuclear localization of NRF2 is indicated with white arrows. **C**–**E** Protein levels of the intranuclear NRF2 and cytoplasmic SGK1 and HO-1 in AAV-NC- or AAV-shSGK1-infected HT22 cells treated with or without dexamethasone. Representative images (**C** and **D**) and statistical analyses (**E**) are shown. *p < 0.05, **p < 0.01 and ***p < 0.001 vs. HT22-NC-Ctrl; ^#^*p* < 0.05, ^##^*p* < 0.01 vs. HT22-NC-Dex; *n* = 3 biologically independent samples. **F** Schematic summary of hypercortisolism-induced neuronal apoptosis synchronized with SGK1-induced neuronal protection in GFAP^+^ neurons in the FrA of mice after SCT.

Moreover, the protective effects of NRF2 signaling are supposed to be mediated through transcriptional regulation of HO-1 expression. Therefore, in addition to protein expression, the intranuclear translocation of the NRF2 protein needs to be examined. Confocal microscopy revealed increased nuclear distribution of NRF2 in HT22 cells after dexamethasone treatment, which could be diminished by the SGK1 silencing mediated by AAV infection (Fig. 8A, B). Similar results of NRF2 nuclear distribution and HO-1 expression activated by dexamethasone-induced SGK1 upregulation and attenuated by SGK1 silencing were also verified by immunoblotting testing cell fractionations of HT22 cells (Fig. 8C−E). These results are consistent with our assumption that enhanced NRF2/HO-1 signaling is dependent upon SGK1 expression in these apoptotic neurons.

## Discussion

The activation, infiltration, and transdifferentiation of neuroglial cells in lesions mainly occur in the subacute phase after SCI, during which microglia and other immunocytes are activated by ischemia-induced cell death [53]. Compared with those of pro-inflammatory microglia, the roles of astrocytes are much more complicated: scar-forming astrocytes in lesions participate in glial scar formation which hinders axonal regrowth; on the other hand, these astrocytes were also found to inhibit further spread of immunocytes and pro-inflammatory cues [54]. Certain oriented differentiation of astrocytes has been reported to shift microglia in lesions towards immune-regulatory phenotypes, thus mitigating post-SCI inflammation [28]. Therefore, we constructed the GFAP-IRES-Venus-AkaLuc KI mouse model to further investigate astrogliosis after SCI. The significant increase in bioluminescence signals in the mouse spinal cords on day 3 after SCT, which corresponds to astrogliosis during the subacute phase, testified the reliability of our mouse model to indicate GFAP expression. At the same time, GFAP expression in mouse heads exhibited unexpected augmentation. The activation of the GFAP gene is vital for the mediation of astrogliosis after CNS injuries and neurodegenerative disorders, and the release of GFAP proteins into biofluids has been receiving increasing attention as a potential neurobiomarker for these diseases [55]. In our SCT model, GFAP upregulation in mouse heads occurred even before the subacute phase (day 1 after SCT), which might suggest potential novel mechanisms other than secondary astrogliosis triggered by cell death. The detection sensitivity of bioluminescence IVI, however, is not precise enough to further locate the exact encephalic regions where post-SCT GFAP upregulation occurs, which prompted us to introduce other research methods.

To date, SCI has been demonstrated to induce neuroinflammation at multiple levels along the neuroaxis, including the cerebrum and brainstem [56]. Specifically, within the cerebrum, research has focused mainly on the post-SCI neuroinflammation and neuronal dysfunction in the basolateral amygdala and hippocampus [43], while the changes in other encephalic regions remain largely elusive. Therefore, we chose to screen for the post-SCI expressional difference of GFAP throughout the whole brain by tissue-clearing techniques with/without antibody staining. Our results showed that post-SCT GFAP upregulation was not detected in either the basolateral amygdala or hippocampus but in other encephalic regions, especially the FrA within the prefrontal cortex. Studies on the FrA are relatively limited and have been reported to be responsible for the consolidation of auditory fear conditioning [52] and associative memory [44]. Dysfunction of the FrA has been reported to mitigate long-term object recognition memory in rodents [45], which is similar to SCI-induced cognitive deficits. More surprisingly, GFAP upregulation in the FrA colocalized with NeuN-positive mature neurons. There have been continuous studies reporting neuronal GFAP expression during the progression of neurological diseases [46, 57]; for example, distinct GFAP splice forms expressed in pyramidal neurons of the hippocampus were identified in the brain tissues of AD patients [46]. Notably, Zwirner et al. [58] recently noted that the neuronal GFAP signals observed in human brains suffering from traumatic brain injury were actually staining artifacts because the anti-GFAP antibody cross-reacted with neurofilament-L [59]. On the other hand, however, the transcription of the GFAP gene in certain neuron clusters was detected by single-cell RNA-sequencing in human brains under both physiological (https://www.proteinatlas.org/ENSG00000131095-GFAP/single+cell+type/brain) and pathological conditions [57, 60]. Here, our findings with this GFAP-IRES-Venus-AkaLuc mouse model contributed to resolving this controversy by applying different techniques to cross-validate GFAP expression in mature neurons after SCT. We hypothesized that this neuronal GFAP expression might hint at certain neuroprotective mechanisms against stress-induced neuronal damage.

Meanwhile, whole-brain tissue from SCT-treated GFAP-IRES-Venus-AkaLuc mice was also subjected to bulk RNA-sequencing. The peroxisome-associated pathway and apoptotic signaling were generated from the GO/KEGG enrichment, which is in accordance with the elevated level of cleaved-Caspase-3. The upregulated DEGs were double-checked with qPCR. Among the significantly upregulated genes cross-validated by qPCR, SGK1 has also been attributed to mediating the regulation of various cerebral functions, including memory consolidation and fear retention [22], which might be associated with the potential physiologic functions of FrA. Thus, the tissue of FrA was segmented and subjected to immunoblotting; the synchronous upregulation of GFAP, SGK1, and biomarkers of cellular apoptosis was observed in SCT-treated wild-type mice. In further in situ detection by immunofluorescence, SGK1 upregulation at the FrA after SCT was confirmed to be mainly localized in GFAP-positive mature neurons as described above, and these are the same neurons suffering from ongoing apoptotic injuries, as indicated by TUNEL/anti-cleaved-Caspase-3 staining.

The induction of SGK1 signaling could be implicated in diverse cellular effects, such as apoptosis resistance, cell cycle acceleration, maintaining mitochondrial homeostasis, and sensitizing various pump, carrier, and membrane channel activities [61–64]. Specifically, the modulation of SGK1 signaling in neurons has been manifested as promoting cell survival and regulating neuronal excitability [22, 65]. It is noteworthy that SGK1 upregulation has been reported to be initiated in response to different stimuli, which can be grouped into two major categories: apoptotic stress, such as cell shrinkage, osmotic shock, and reactive oxygen species (ROS), or hormones, such as mineralocorticoids and glucocorticoids [22, 23]. Therefore, the exact inducers of SGK1 upregulation we discovered in GFAP-positive neurons within the FrA of SCT-treated mice, which are under apoptotic stress, need to be further specified. Endogenous glucocorticoids bind to both MR and GR for subsequent transcriptional regulation; however, due to their higher affinity for glucocorticoids, MR is usually fully occupied even under physiological conditions [27]. These facts directed our attention to exploring the expression and distribution of GR-α within the cerebral microenvironment, which is known as the major isoform of GR involved in transcriptional regulation [66]. The results showed that the GR-α protein was mainly expressed in mature neurons within the mouse FrA, which turned into GFAP-positive and was under apoptotic stress after SCT surgery. On the other hand, the serum levels of cortisol remained above the basal levels until at least day 7 after SCT, while the expression of GR-α in these neurons did not decrease (there was no glucocorticoid resistance) [67]. These results were consistent with our assumption that hypercortisolism under acute stress leads to excessive amounts of glucocorticoids penetrating the blood-brain barrier, exerting neurotoxic effects on neurons in the cerebral cortex; the main functional mechanism could be attributed to cellular apoptosis [68]. After introducing sustained treatment with GR antagonist mifepristone to the SCT-treated mice [31, 69], the upregulation of SGK1 within the FrA after SCT was partially reversed. That is, the persistent increase in glucocorticoid levels within the CNS is at least partially accountable for SGK1 upregulation in these GR-positive mature neurons undergoing apoptosis in the FrA of SCT-treated mice, which is also in line with the findings of Sato et al. and Sarabdjitsingh et al. about the role of glucocorticoids in regulating cerebral SGK1 expression [70, 71].

However, the fact that the upregulation of anti-apoptotic SGK1 is synchronized with glucocorticoid-induced neuronal apoptosis raised our doubts about the exact regulatory roles of SGK1 signaling during this process. AAV vectors designated for neuron-specific SGK1 silencing were constructed, verified, and injected specifically into the FrA before SCT surgery. It turned out that SGK1 knockdown further enhanced cellular apoptosis in the FrA of SCT-treated mice; correspondingly, in the extensively studied in vitro model of dexamethasone-treated HT22 neurons [32, 33], successful SGK1 knockdown was also found to promote dexamethasone-induced apoptosis. Taken together, the SGK1 upregulation we discovered in GFAP-positive neurons within the FrA participates in the mediation of apoptosis resistance but is insufficient to fully inhibit the apoptosis process initiated by excessive cerebral glucocorticoids after SCT. Moreover, GFAP expression after SCT varies in different encephalic regions; that is, the GFAP-positive neurons in the FrA are region-specific, and this GFAP expression is positively correlated with neuronal apoptosis regulated by glucocorticoids and SGK1 expression. We have not deciphered the exact mechanisms by which these neurons express GFAP, which could be better elaborated in the future with more advanced research tools, such as the combination of spatial transcriptome and single-nucleus RNA-sequencing [72, 73].

Additionally, we also explored the downstream functioning mechanisms of upregulated SGK1 in these GFAP-positive neurons. SGK1-mediated apoptosis resistance has been canonically reported to be effectuated through the regulation of Foxo3a signaling [49, 74], which was also consistent with the FoxO signaling pathways generated from KEGG enrichment of the bulk RNA-sequencing testing the whole brains of SCT-treated mice in this study (Fig. S2). In addition, the ability of SGK1 to promote cell survival has also been attributed to antioxidative effects related to the expression of NRF2-associated genes [59, 75]. NRF2 is considered to be activated under oxidative conditions [76], playing a central role in maintaining redox balance and resisting ROS injuries [77] by subsequently regulating the transcription of oxidoreductases and oxygenases such as HO-1 [78]. Multiple studies have demonstrated that the activation of NRF2/HO-1 signaling attenuates neuronal apoptosis [79–81], yet its relationship with SGK1 has not been fully elucidated. Here, enhanced NRF2 and HO-1 expression was detected in the FrA of SCT-treated mice, both of which could be partially reversed by mifepristone pretreatment or AAV-mediated SGK1 knockdown. Moreover, subcellular colocalization and nuclear extraction were applied to dexamethasone-treated apoptotic HT22 cells. Both assays uniformly confirmed that SGK1 upregulation under dexamethasone treatment was accompanied by increased intranuclear distribution of NRF2, which could be mitigated by SGK1 silencing. Hence, we concluded NRF2/HO-1 axis as a noncanonical downstream signaling pathway of SGK1 activation in apoptotic neurons within the FrA of SCT-treated mice.

There is no direct neural connection between the spinal cord and the FrA. The fact that neuronal injuries discovered in the FrA were induced by sustained hypercortisolism after SCT revealed the importance of intercommunication between the CNS and endocrine system, as well as feedback regulation of the HPA axis [24]. On the other hand, studies on SCI have been largely driven by the demands to improve injury repair and ameliorate post-SCI emotional disorders and motor dysfunctions [7, 82–85]. Our evaluations about introducing exogenous SGK1 intervention into these GFAP-positive FrA neurons showed no significant benefits on the hindlimb locomotion of mice suffering from SCI. Meanwhile, SGK1 intervention in these FrA neurons did aggravate the post-SCI depressive-like behaviors, which indicates the potential for both SGK1 and FrA neurons as therapeutic targets to cure post-SCI emotional disorders. More sophisticated tests are required to further verify this notion. However, the following questions remain to be addressed in this study: it remains unknown whether GFAP-positive neurons in encephalic regions other than FrA are apoptotic; the phenotypes and functions of apoptotic GFAP-positive neurons in the FrA are unclear; the exact mechanisms underlying their sensitivity to high-level glucocorticoids are largely elusive since GR-α expression is not restricted, but rather widely distributed in neurons within the entire limbic nervous system, including the basolateral amygdala and hippocampus (Fig. S14) [86]. These issues could be among our future research orientations.

## Conclusion

Overall, this study for the first time revealed the presence of apoptotic neurons located in the FrA, which become GFAP-positive after SCI with the help of the GFAP-IRES-Venus-AkaLuc mouse model. Briefly, SCT surgery leads to hypercortisolism in mice for stress reactions; excessive glucocorticoids enter the CNS and bind to the GR located in the neurons in the FrA, which activates neuronal apoptosis and SGK1 expression; SGK1 exerts neuroprotective effects via activating NRF2/HO-1 signaling. However, these regulatory effects were not sufficient enough to restrain the progression of apoptosis in glucocorticoid-stimulated neurons (Fig. 8F). These findings emphasized the roles of SGK1 activation, which might provide new insights for the development of therapeutic strategies for spinal cord injuries. For instance, combining the exogenous SGK1 expression and the introduction of GR inhibitors might be a potential method to utilize SGK1-mediated neuronal protection while preventing glucocorticoid-induced apoptotic signaling, thus mitigating neuronal death after SCI.

## Supplementary information

Revised Supplementary

Uncropped IB blots

Video. S1 Lower limb motor dysfunction of SCT-treated mouse

Video. S2 Lower limb tactile dysfunction of SCT-treated mouse

Video. S3 3D-reconstruction of GFAPXNeuN stained mouse brain after tissue clarity

## Data Availability

The data will be made available upon request.
