# Supplementary material for: SGK1 upregulation in GFAP+ neurons in the frontal association cortex protects against neuronal apoptosis after spinal cord injury
Source: Cell Death Dis. 2025 Apr 2;16(1):237. doi: 10.1038/s41419-025-07542-y (PMC11965300; doi:10.1038/s41419-025-07542-y)
Supplement: Supplementary file 1 — Revised Supplementary [file 41419_2025_7542_MOESM1_ESM.docx]

**Supplemental Tables and Figures**

**Supplemental Table 1. Primer sequences for qPCR**

| **Gene** | Forward (5’ to 3’) | Reverse (5’ to 3’) |  |
| --- | --- | --- | --- |
| Mouse *Hif3α* | gaagttcacatactgcgacga | gtccaaagcgtggatgtattcat |  |
| Mouse *Sgk1* | ctgctcgaagcacccttacc | tcctgaggatgggacattttca |  |
| Mouse *Plin4* | gtgtccaccaactcacagatg | ggaccattccttttgcagcat |  |
| Mouse *Sult1a1* | caacatggagcccttgcgtaa | atgagcacatcatcaggccag |  |
| Mouse *Map3k6* | gcctctcagtgtggtcatcg | cgtcgcaaagggtaggctg |  |
| Mouse *Efcab7* | aaccctggaagtgatgcagc | gtaggcggctctacagttcat |  |
| Mouse *Dsg3* | tggcagtctggaagtcacc | ctgtagagggtcagggatgg |  |
| Mouse *Abcc4* | aggagcttcaacggtactgg | gcctttgttaaggagggcttc |  |
| Mouse *Fga* | agtctggactacagataccgaag | cgtcaatcaaccctttcatcctg |  |
| Mouse *Xdh* | atgacgaggacaacggtagat | tcatacttggagatcatcacggt |  |
| Mouse *Pla2g3* | | agagaccacagggccattaag | gctgtagaatgacatggtgct |
| Mouse *Lrg1* | | ttggcagcatcaaggaagc | cagatggacagtgtcggca |
| Mouse *Serpina3f* | | tgggagatgccctttgatcc | agtagggtgtcgtcaggttatta |
| Mouse *Steap4* | | gggaagtcactgggattgaaaa | ccgaatagctcaggacctctg |
| Mouse *Tekt4* | | ggccccacagtcaatagatgt | gggcataagagttctgaaacca |
| Mouse *Spata48* | gccagccaaaccaatcaattt | ttgtccacattgtcagcgtct |  |
| Mouse *Apoa1* | ggcacgtatggcagcaagat | ccaaggaggaggattcaaactg |  |
| Mouse *Fancd2os* | accccactggatgagagctt | gaaggcacaactgcacttcaa |  |
| Mouse *Mefv* | tcatctgctaaacaccctgga | gggatcttagagtggcccttc |  |
| Mouse *Cyp4a10* | ttccctgatggacgctcttta | gcaaacctggaagggtcaaac |  |
| Mouse *Gfap*-pair1 | ccctggctcgtgtggattt | gaccgataccactcctctgtc |  |
| Mouse *Gfap*-pair2 | cggsgscgcatcacctctg | agggsgtggaggagtcattcg |  |
| Mouse *Gapdh* | aggtcggtgtgaacggatttg | tgtagaccatgtagttgaggtca |  |

**Supplemental Table 2. Antibodies for assays**

| **Antibody** | **Vendor and Cat. No.** | | **Application** |  |
| --- | --- | --- | --- | --- |
| Mouse anti-GFAP | Cell Signaling Technology, 3670S | | IF/IHF (1:500);  TC (1:1000) |  |
| Rabbit anti-GFAP | Proteintech, 16825-1 | | IB (1:2000) |  |
| Mouse anti-p53 | Abcam, ab26 | | IB (1:2000) |  |
| Rabbit anti-GSDMD | Novus, NBP2-33422 | | IB (1:800) |  |
| Rabbit anti-phospho-MLKL (Ser345) | Novus, NBP2-66953 | | IB (1:800) |  |
| Rabbit anti-LC3 | Abcam, ab192890 | IB (1:800) | |  |
| Rabbit anti-p62 | MBL, PM045 | IB (1:800) | |  |
| Rabbit anti-GPX4 | Cell Signaling Technology, 52455S | IB (1:800) | |  |
| Rabbit anti-caspase3 | Cell Signaling Technology, 9662S | IHF (1:200) | |  |
| Rabbit anti-cleaved caspase3 (Asp175) | Cell Signaling Technology, 9661S | IB (1:800) | |  |
| Rabbit anti-cleaved caspase3 | R&D, AF835 | IHF (1:200) | |  |
| Mouse anti-NeuN | Proteintech, 66836-1 | IHF (1:200) | |  |
| Rabbit anti-NeuN | Cell Signaling Technology, 24307S | IHF (1:400);  TC (1:1000) | |  |
| Rat anti-NeuN | Oasis biofarm,  OB-PRT013-01 | IHF (1:400) | |  |
| Rabbit anti-SGK1 | ABclonal, A1025 | IF/IHF (1:50) | |  |
| Rabbit anti-SGK1 | Wanlei, WL02741 | IB (1:800) | |  |
| Mouse anti-Myc tag | Origene, TA183053 | IB (1:500) | |  |
| Rabbit anti-Glucocorticoid receptor alpha | Invitrogen, PA1-516 | IHF (1:200) | |  |
| Rabbit anti-Glucocorticoid receptor (alpha + beta) | Cell Signaling Technology, 12041 | IHF (1:200) | |  |
| Mouse anti-GAPDH | ZSGB-Bio, TA-08 | IB (1:2000) | |  |
| Mouse anti-β-actin | ZSGB-Bio, TA-09 | IB (1:2000) | |  |
| Rabbit anti-NRF2 | Proteintech, 16396-1 | IB (1:1000) | |  |
| Rabbit anti-HO1 | | Cell Signaling Technology, 86806 | IB (1:800) | |
| Rabbit anti-Lamin B | Proteintech, 12987-1 | IB (1:800) | |  |
| Rabbit anti-Histone H3 | Cell Signaling Technology, 4499S | IB (1:800) | |  |
| Goat anti-rabbit IgG-HRP | Cell Signaling Technology, 7074S | IB (1:4000) | |  |
| Horse anti-mouse IgG-HRP | Cell Signaling Technology, 7076S | IB (1:4000) | |  |
| Goat anti-mouse IgG-  Alexa Fluor 488 | Abcam, ab150113 | IF/IHF (1:500) | |  |
| Goat anti-mouse IgG-  Alexa Fluor Plus 555 | Abcam, ab150114 | IF/IHF (1:500);  TC (1:1000) | |  |
| Goat anti-rabbit IgG-  Alexa Fluor Plus 555 | Abcam, ab150078 | IF/IHF (1:500) | |  |
| Goat anti-rabbit IgG-  Alexa Fluor 647 | Abcam, ab150083 | IHF (1:500);  TC (1:1000) | |  |
| Goat anti-rat IgG-  Alexa Fluor 647 | Abcam, ab150167 | IF/IHF (1:500) | |  |
| Goat anti-rabbit IgG  -Alexa Fluor Plus 488 | Abcam, ab150077 | IF/IHF (1:500) | |  |

IB, immunoblotting; IF, immunofluorescence; IHF, immunohistofluorescence; TC, tissue clarity with antibody staining.

**Supplemental Figure 1**

**
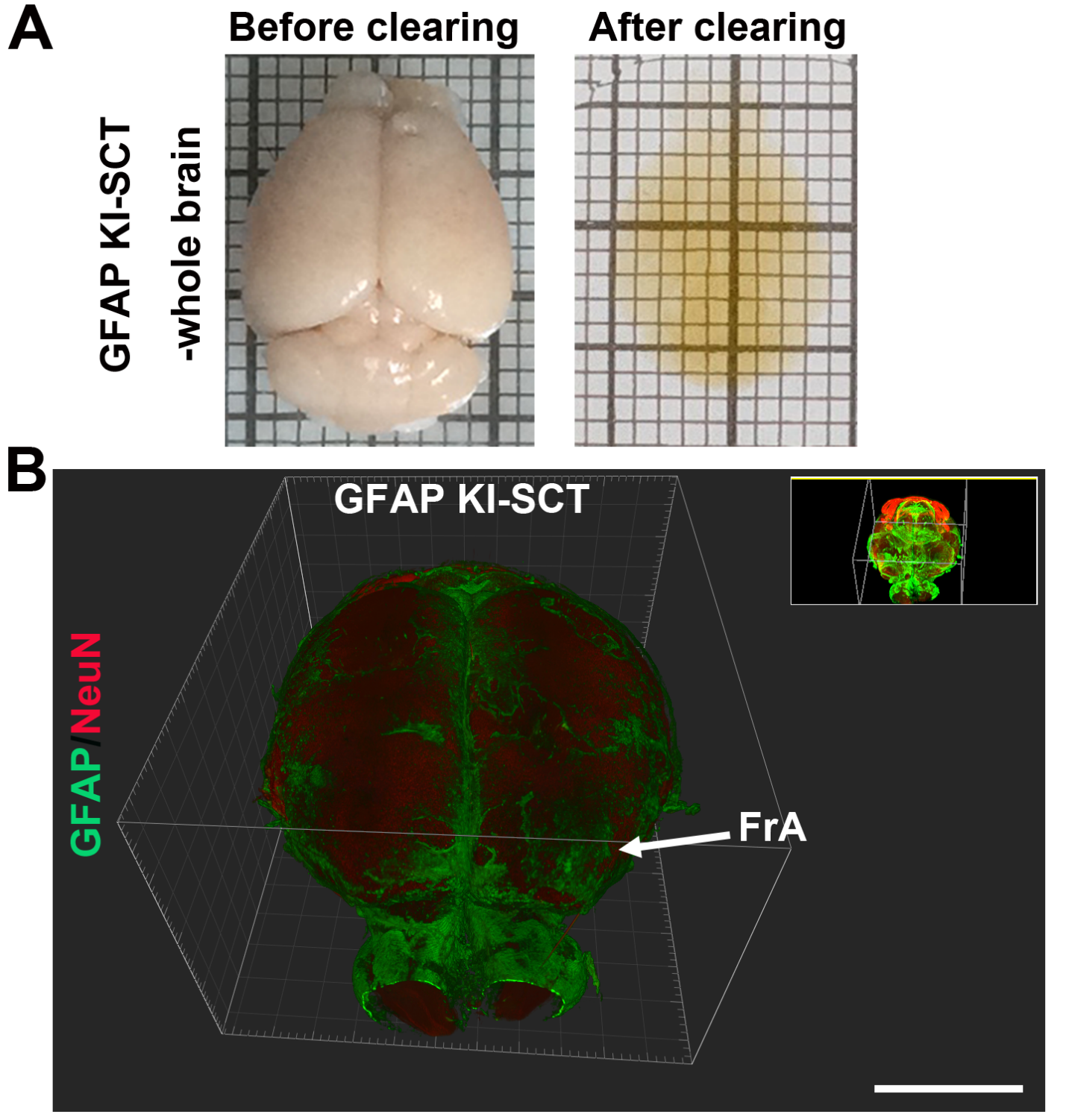
**

**Supplemental Fig. 1 (A)** Representative images of the whole brain from a SCT-treated KI mouse before and after the tissue clarity procedures with antibody staining. **(B)** Representative 3D-reconstructed images of the whole brain from a SCT-treated KI mouse after staining with anti-GFAP (green)/anti-NeuN (red) and tissue clearing. The frontal association cortex (FrA) was indicated with white arrows. Scale bar, 4000 μm.

**Supplemental Figure 2**

**
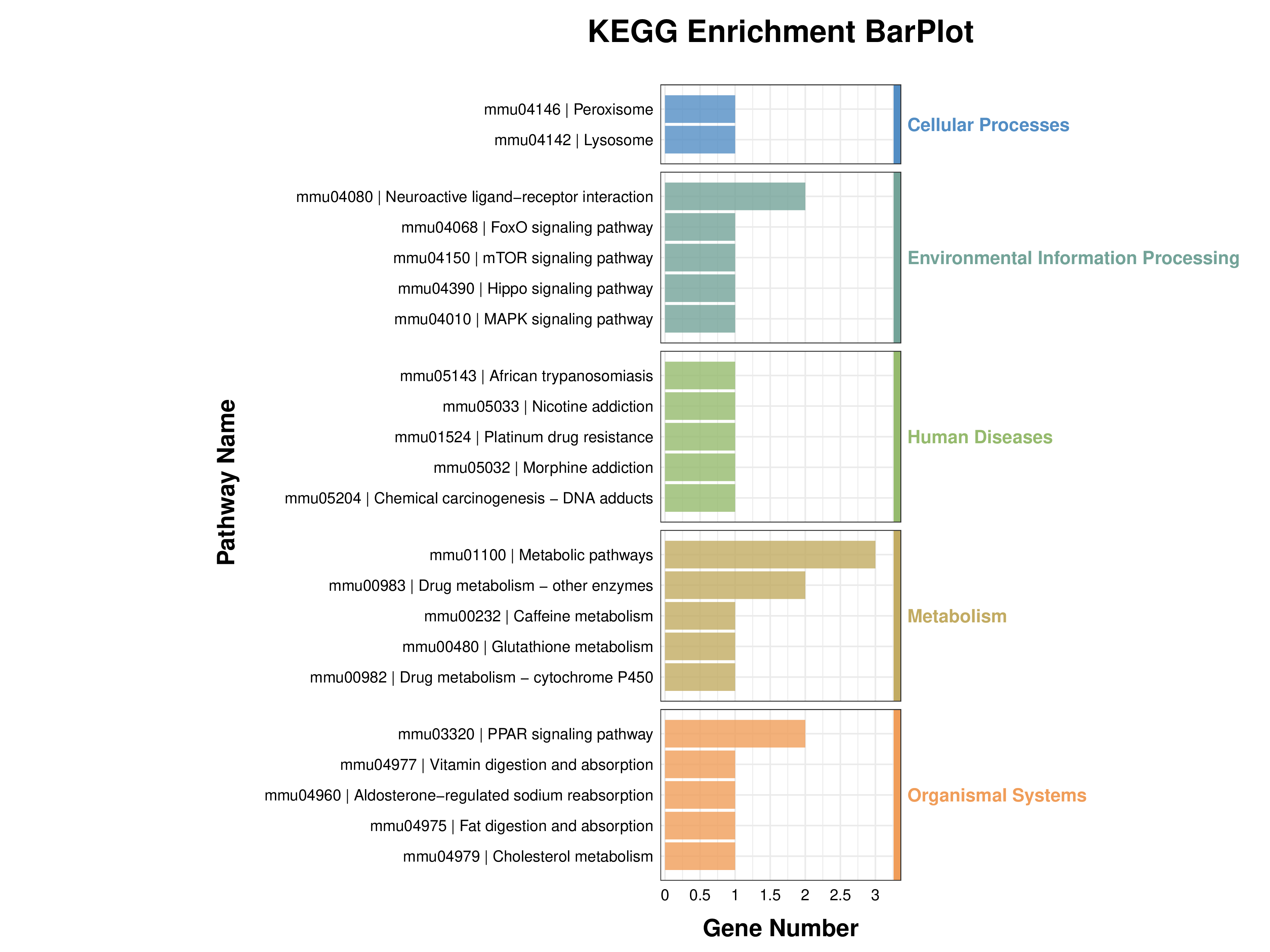
**

**Supplemental Fig. 2** Representative KEGG enrichment bar plot of the bulk-sequencing data of whole brain samples harvested from SCT-treated mice 7 days after surgery. The reported upstream/downstream pathways of SGK1, namely FoxO signaling and mTOR signaling, were enriched according to the bulk-sequencing results.

**Supplemental Figure 3**

**
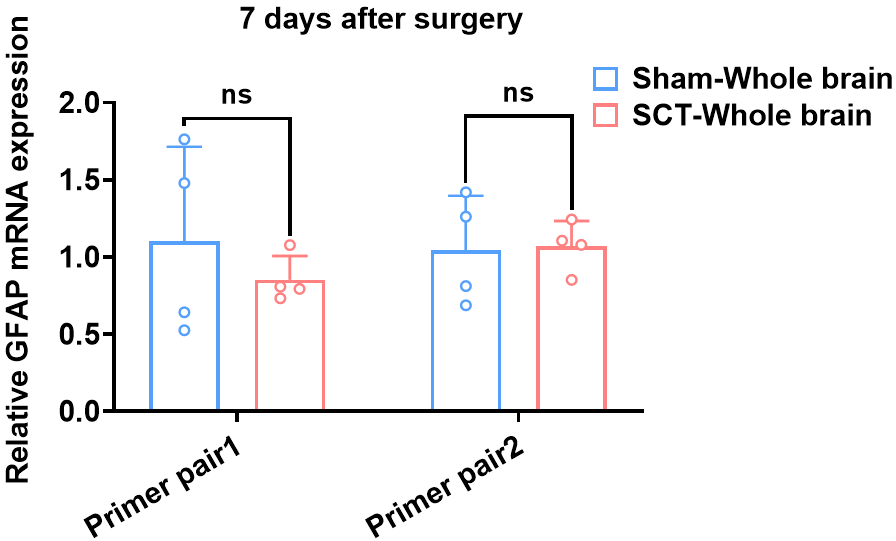
**

**Supplemental Fig. 3** qPCR detection of *Gfap* mRNA level in the whole brain of sham- or SCT-treated KI mice with two primer pairs.

**Supplemental Figure 4**

**
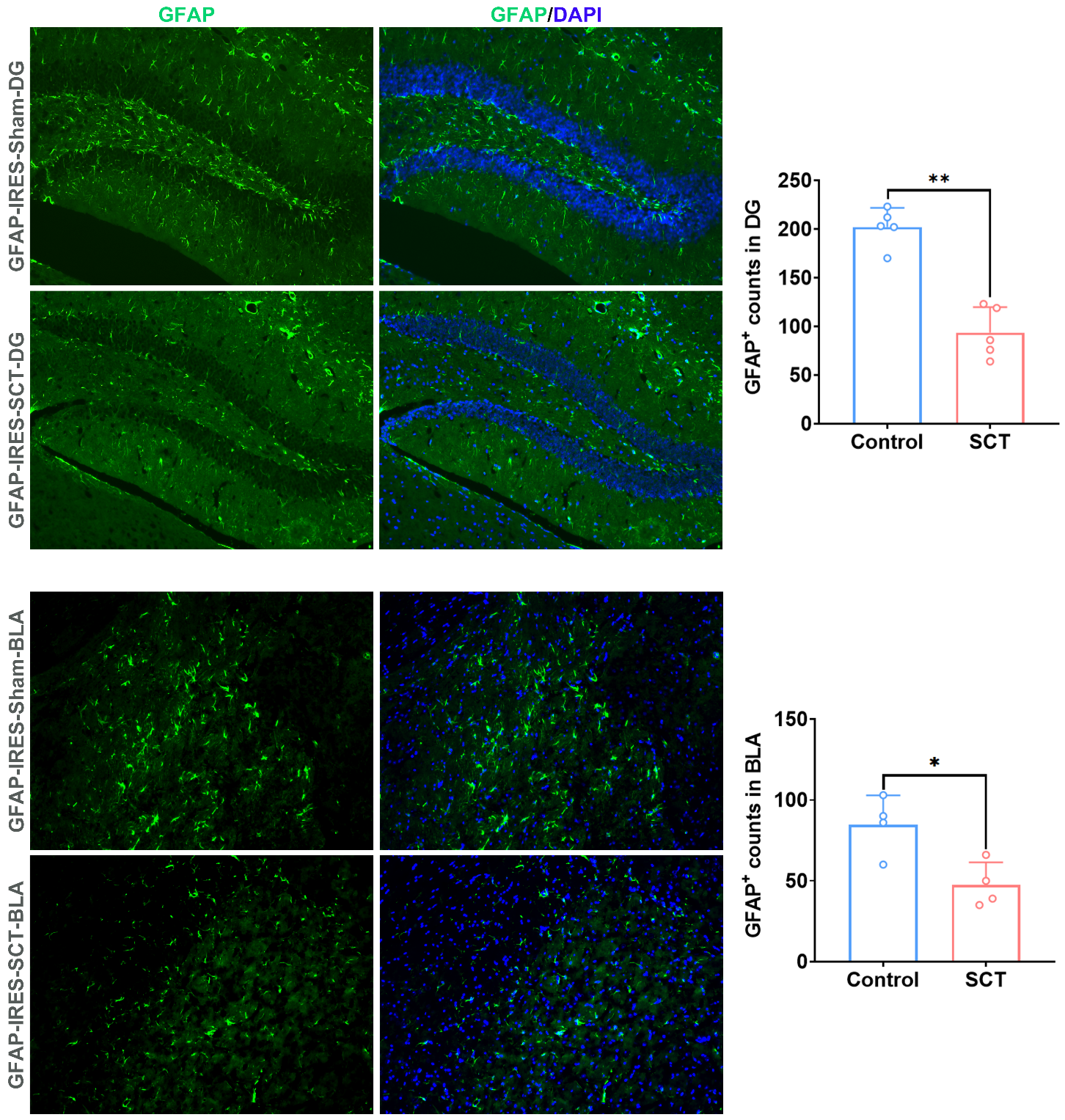
**

**Supplemental Fig. 4** Coronal sections of the hippocampus dentate gyrus (DG) and basolateral amygdala (BLA) from SCT-treated, GFAP-IRES-Venus-AkaLuc KI mice or negative controls treated with sham surgery were stained with anti-GFAP (green); the corresponding GFAP^+^ counts within the DG and BLA were analyzed (*p<0.05, **p<0.01 vs. Sham).

**Supplemental Figure 5**

**
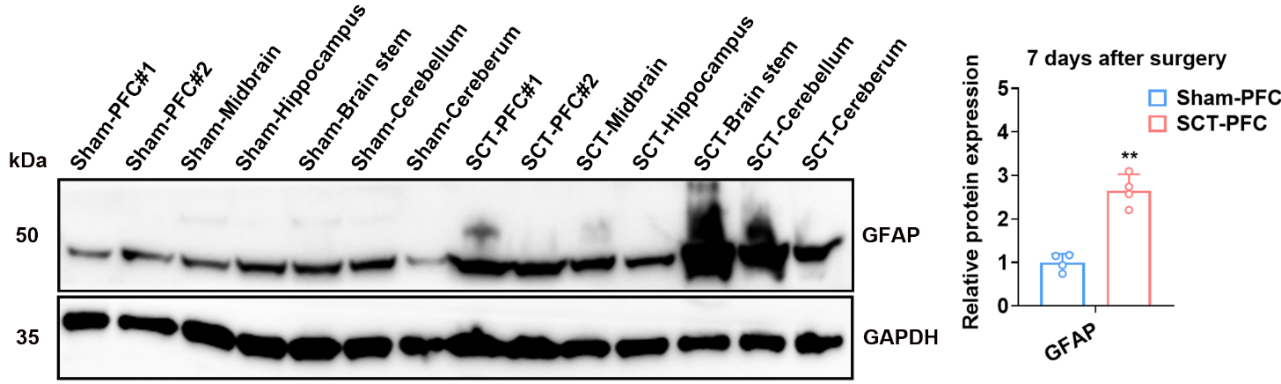
**

**Supplemental Fig. 5** Protein expression of GFAP in various encephalic regions of SCT-treated KI mice 7 days after surgery. GFAP was significantly upregulated in the prefrontal cortex of SCT-treated mice (**p<0.01 vs. Sham).

**Supplemental Figure 6**

**
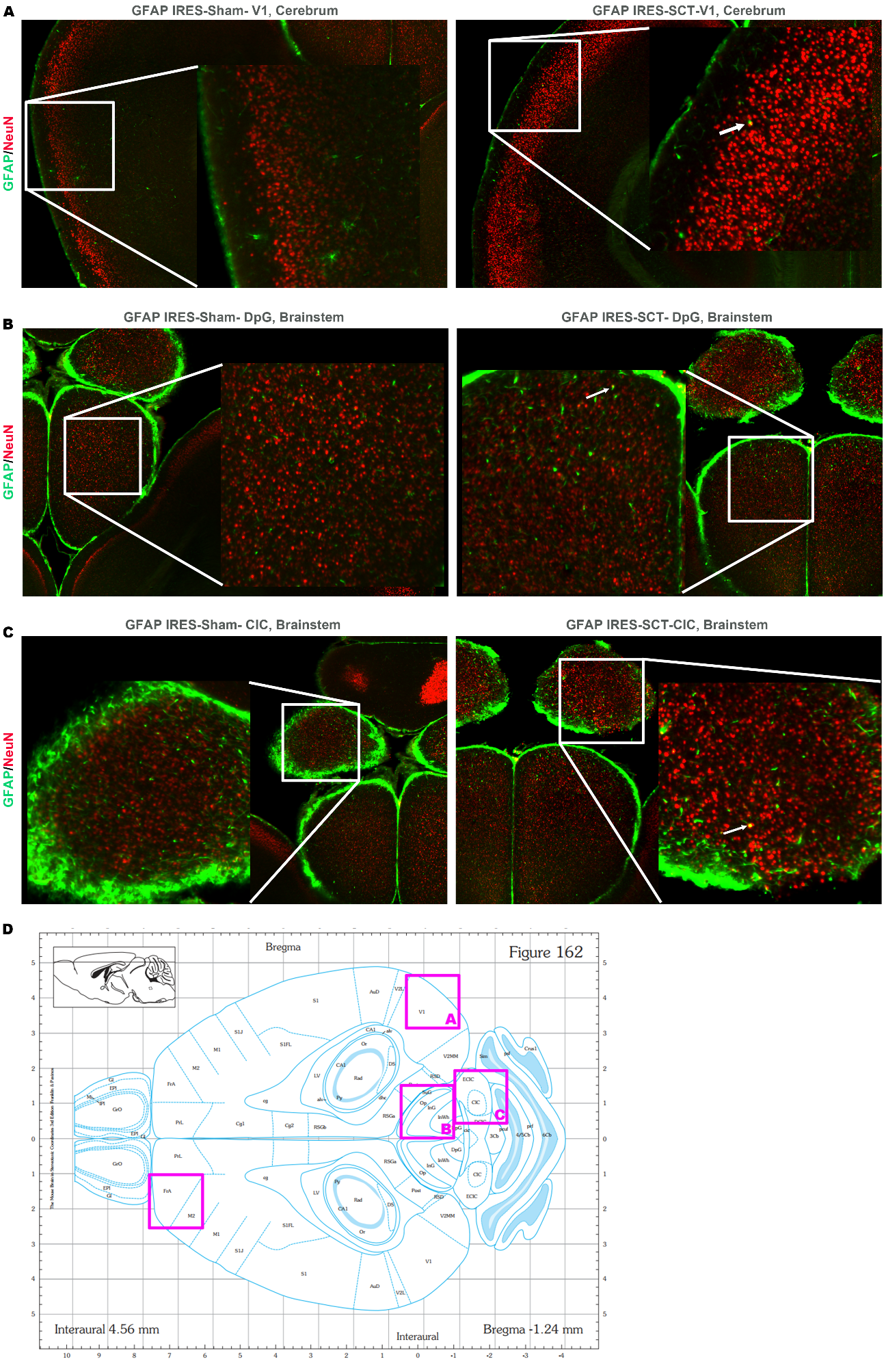
**

**Supplemental Fig. 6** Representative transverse section images of the whole brain of a SCT-treated KI mouse and its sham surgery control after immunostaining-compatible tissue clearing (iDISCO) with antibodies against GFAP (green) and NeuN (red). The GFAP^+^NeuN^+^ cells were discovered in primary visual cortex (V1) of the cerebrum **(A)**, as well as areas around central nucleus of the inferior colliculus (CIC) and dorsal paragigantocellular nucleus (DpG) of the brain stem **(B, C)**. Typical GFAP^+^NeuN^+^ cells were indicated with white arrows. **(D)** All the regions where GFAP^+^ NeuN^+^ cells were discovered in this study were indicated by magenta squares in transverse sections of mouse brain.

**Supplemental Figure 7**

**
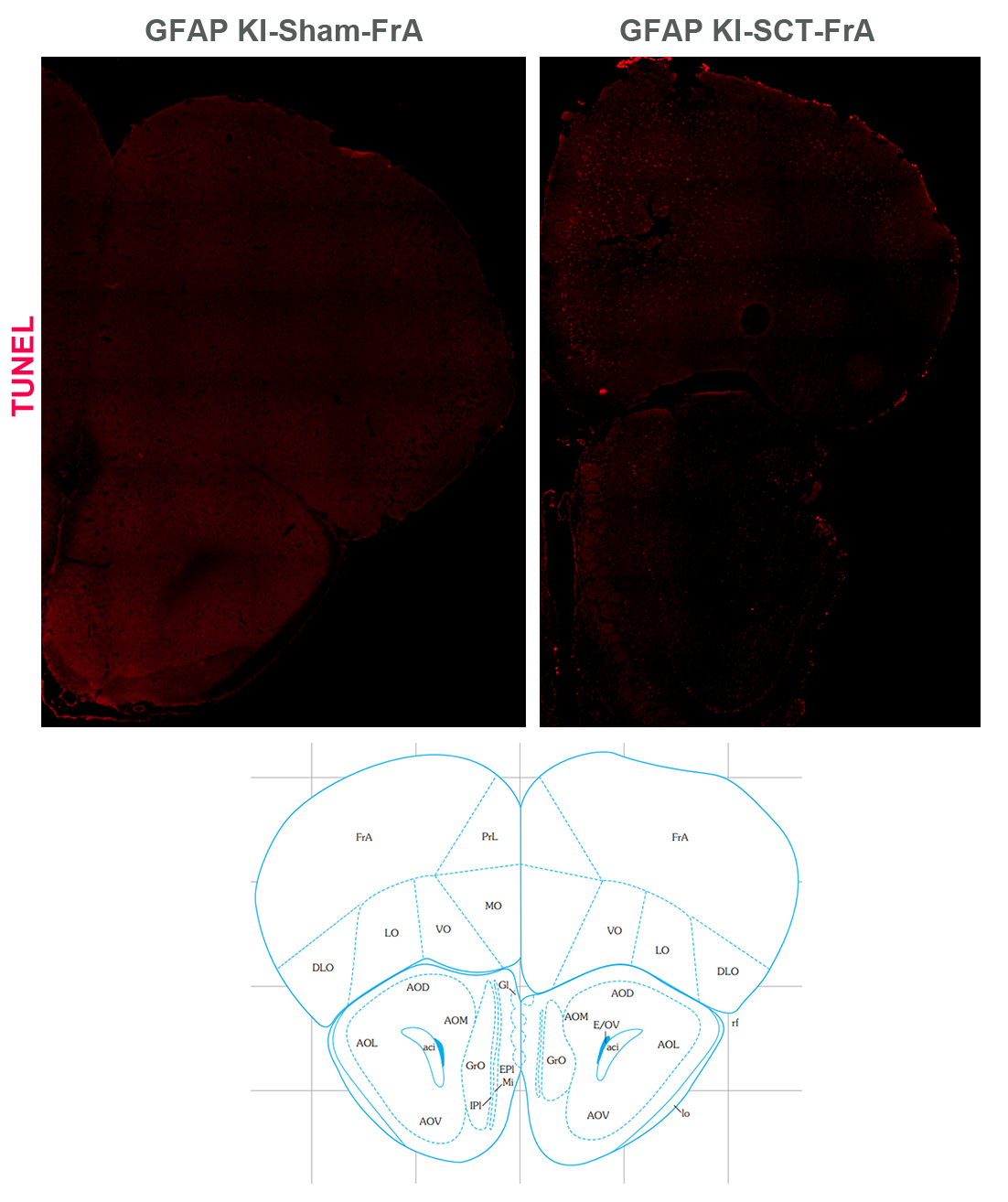
**

**Supplemental Fig. 7** Representative immunofluorescence images of coronal sections of the prefrontal cortex from SCT-treated KI mice were stained with TUNEL-AF555 probe. TUNEL^+^ signals were mainly localized in the FrA within the prefrontal cortex of SCT-treated mice.

**Supplemental Figure 8**


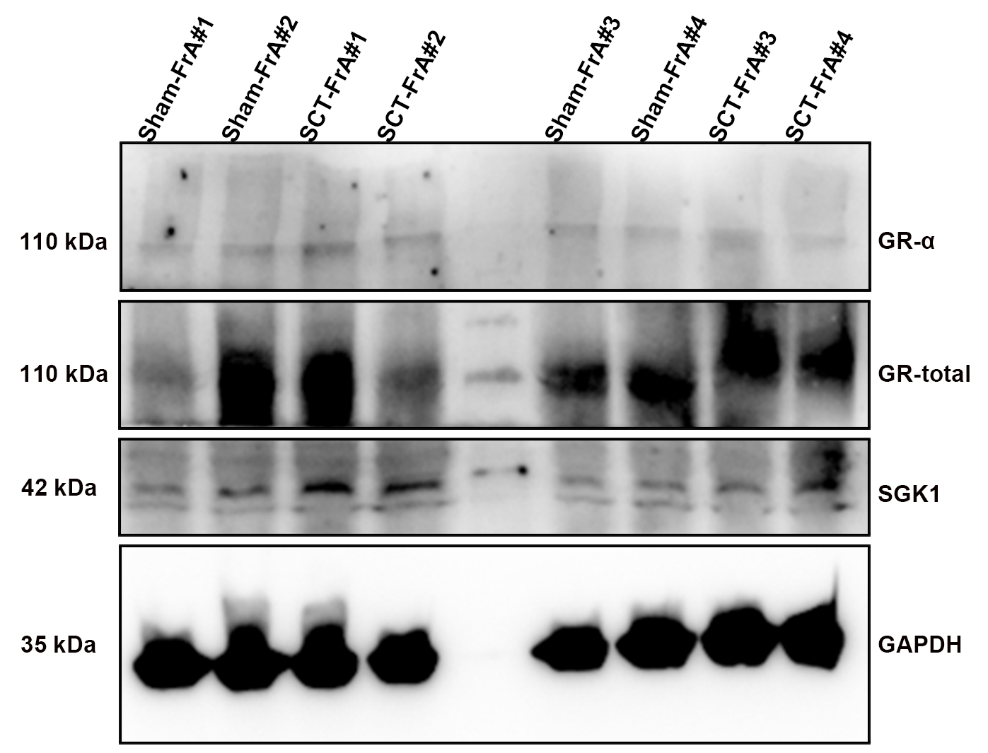


**Supplemental Fig. 8** Protein expression of GRα, total GR and SGK1 in the FrA from SCT-treated mice 7 days after surgery. The results showed that the protein levels of GRα and total GR were not affected by SCT.

**Supplemental Figure 9**


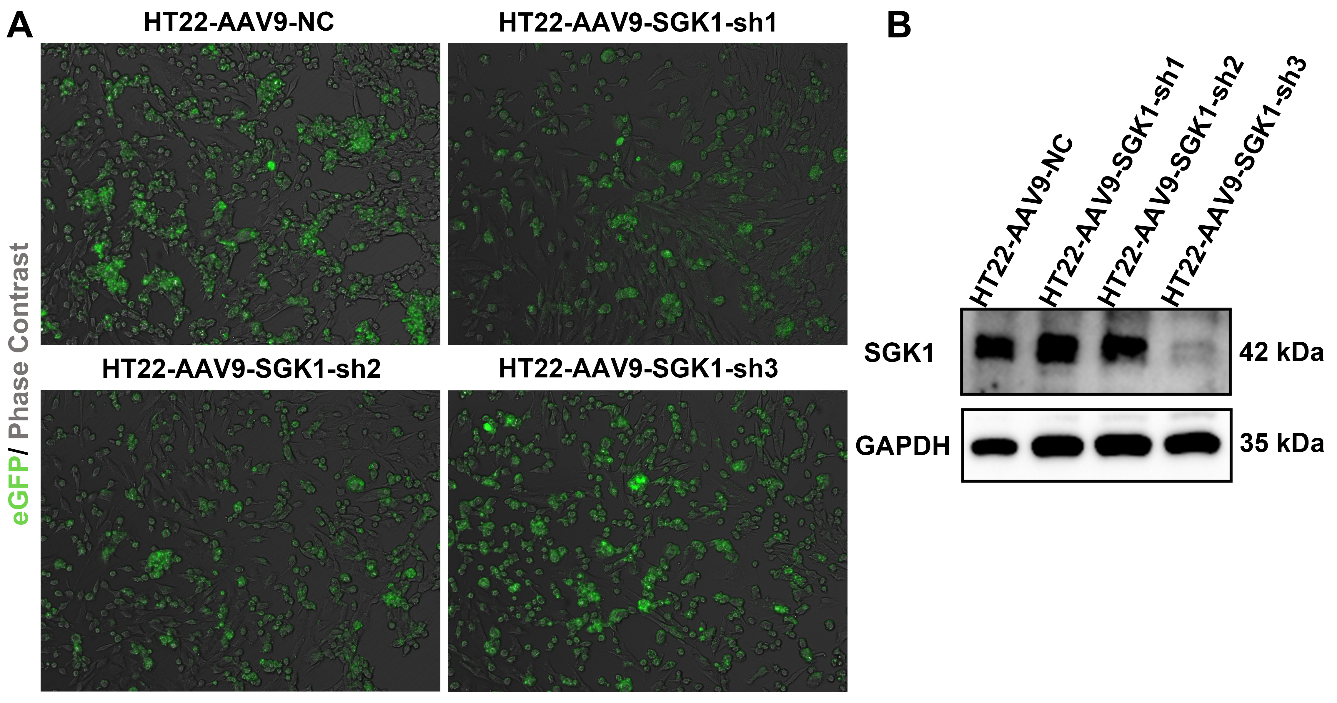


**Supplemental Fig. 9** **(A)** Immunofluorescence images indicating the infection efficiencies of adeno-associated virus (AAV) in mouse hippocampal neuron cell line HT22. **(B)** Immunoblotting testing the *in vitro* knockdown efficiencies of different shSGK1 sequences enveloped into AAV applied in **Fig. S9A**.

**Supplemental Figure 10**


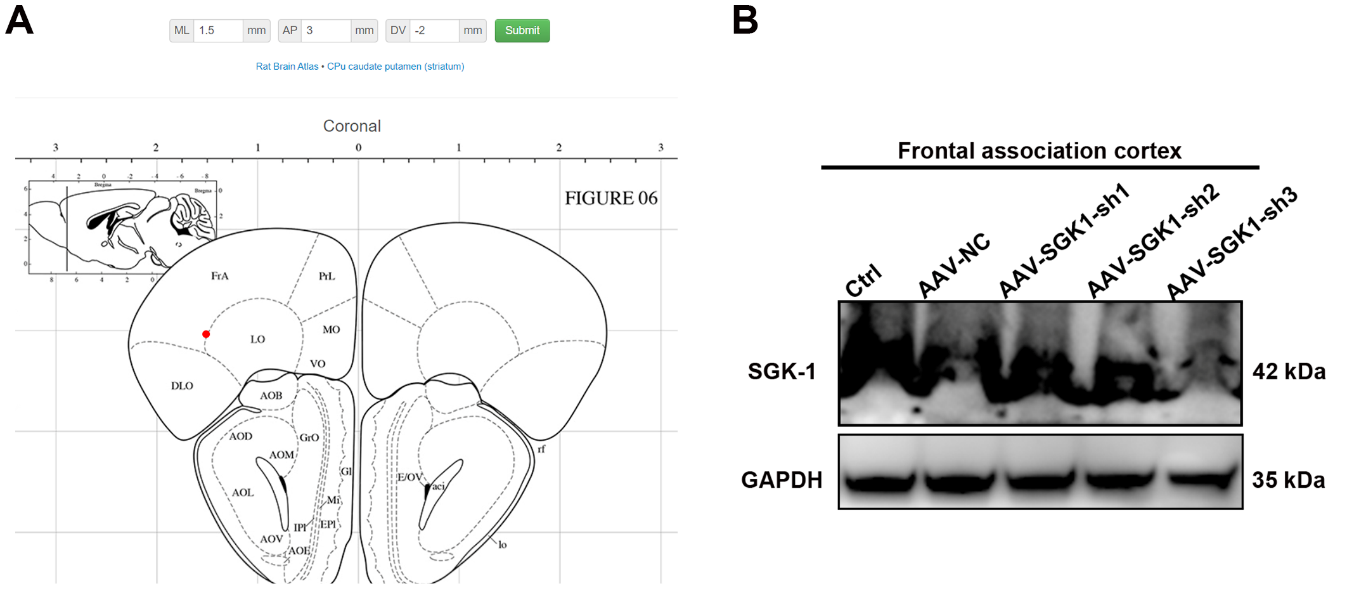


**Supplemental Fig. 10** **(A)** The injection site of AAV was set based on the stereotaxic coordinates from the Mouse Brain Atlas (mediolateral/ML=1.5 mm, anteroposterior/AP=3 mm, dorsoventral/DV=-2 mm). **(B)** Immunoblotting testing the *in vivo* knockdown efficiencies of different shSGK1 sequences enveloped into AAV applied in **Fig. S10A**.

**Supplemental Figure 11**


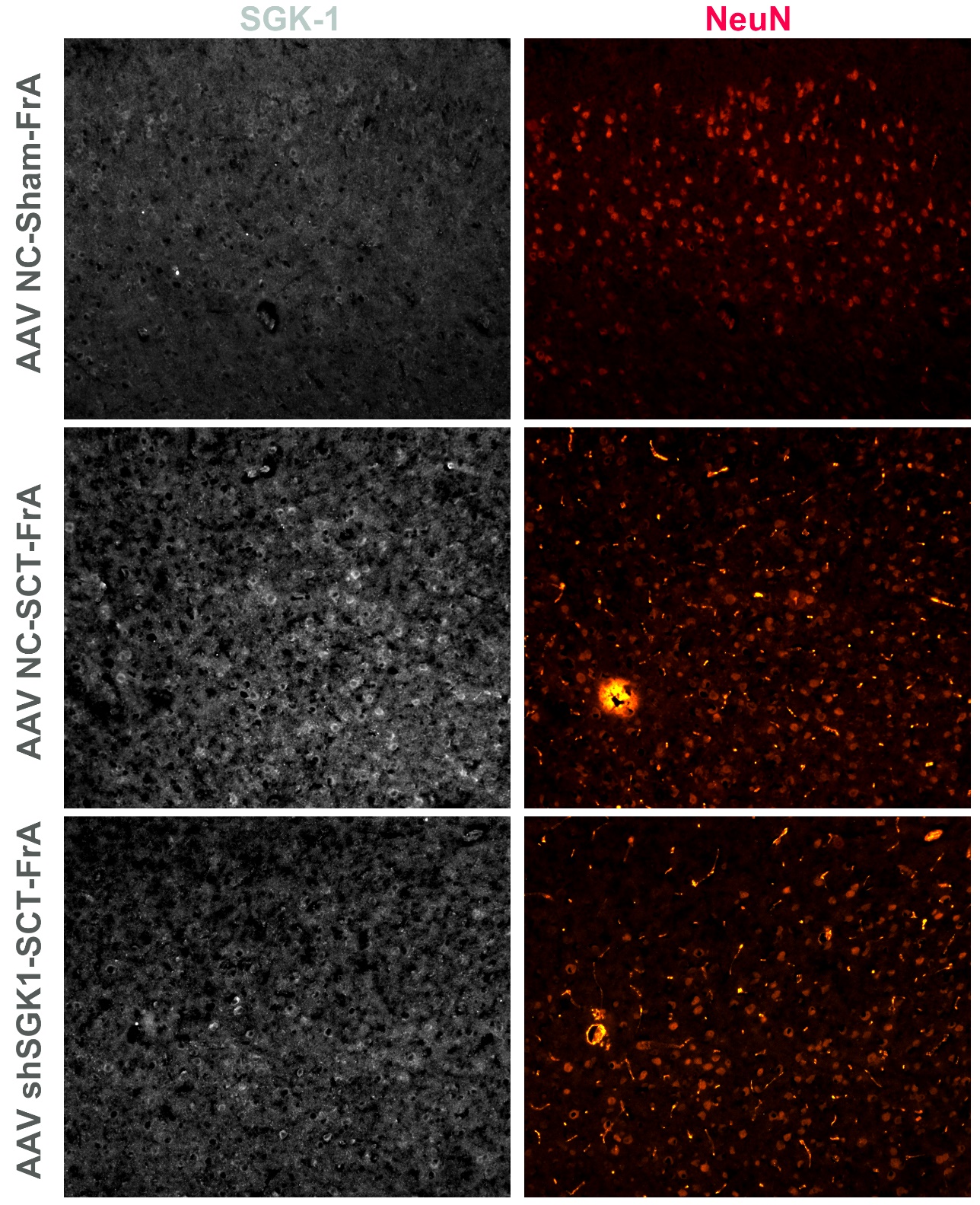


**Supplemental Fig. 11** Coronal sections of the FrA from SCT-treated mice with or without AAV injection prior to SCT surgery were costained with anti-SGK1 (white) and anti-NeuN (red). The results verified that the knockdown of SGK1 at the FrA implemented with the infection of AAV-shSGK1 (Fig. S10B) corresponds to the neuron-specific knockdown of SGK1 upregulation after SCT surgery, which is also in accordance with the immunoblotting results in Fig. 6C.

**Supplemental Figure 12**


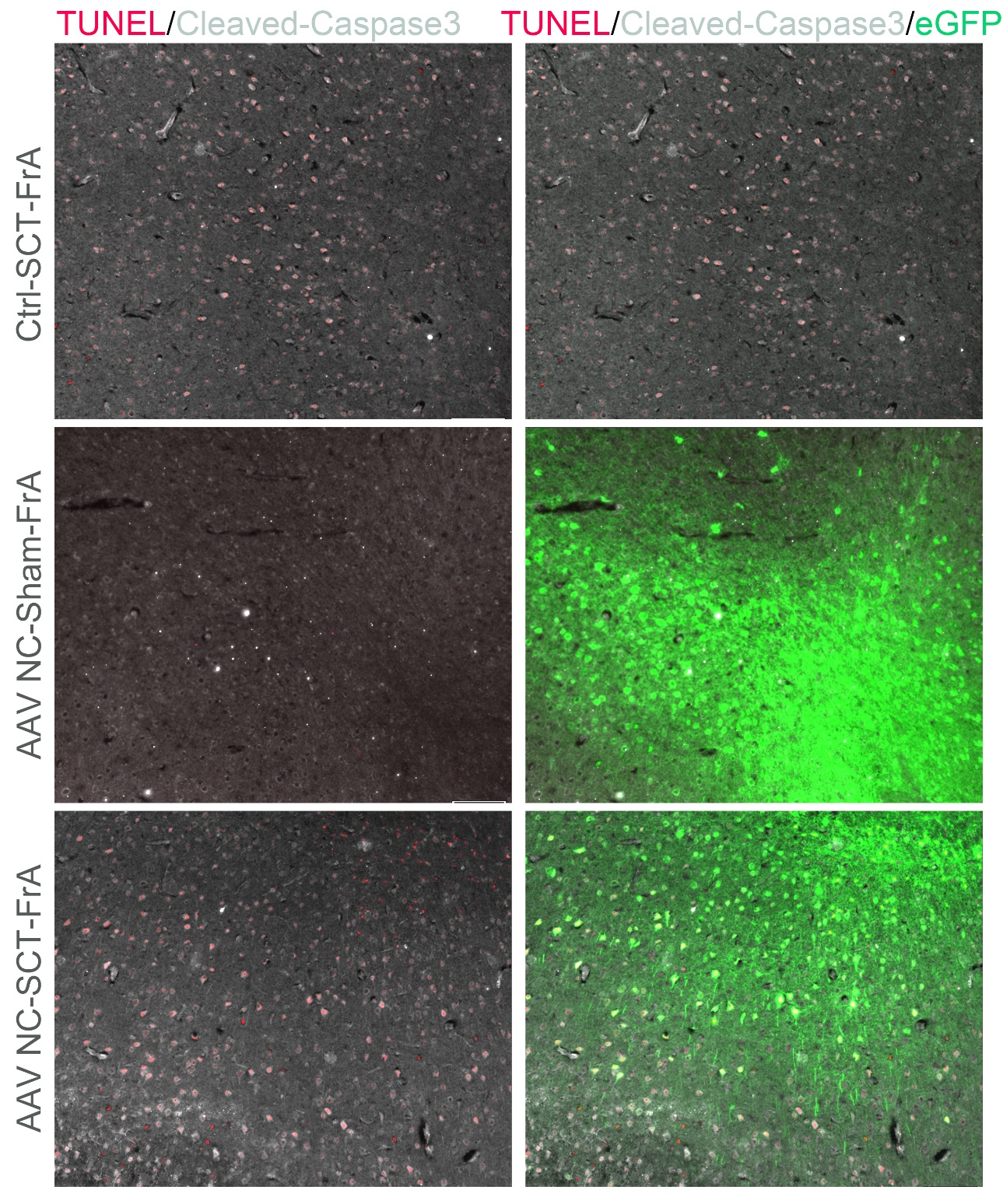


**Supplemental Fig. 12** Coronal sections of the FrA from SCT-treated mice with or without AAV injection prior to SCT surgery were costained with anti-cleaved caspase3 and TUNEL probes. The results showed that the injection of empty AAV vectors alone did not cause neuronal apoptosis at the injection site; on the other hand, neuronal apoptosis at the FrA did not interfere with the infection and expression of injected AAV.

**Supplemental Figure 13**


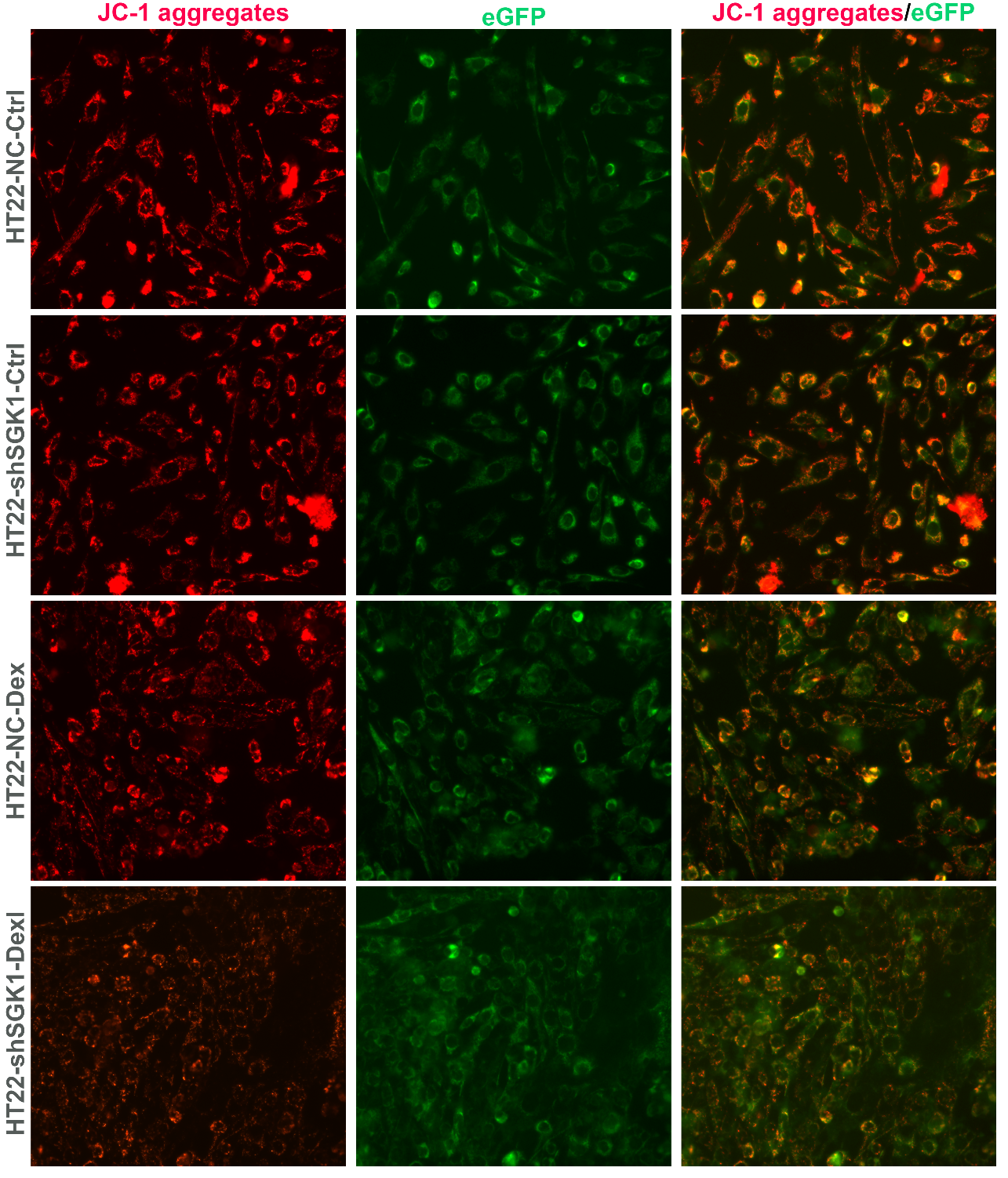


**Supplemental Fig. 13** The mitochondrial membrane potentials of dexamethasone-treated HT22 cells after the infection with AAV-shSGK1 were measured using a JC-1 fluorescence probe. Diminished signals of JC-1 aggregates in dexamethasone-treated HT22 cells indicated decreased mitochondrial membrane potentials. Knockdown of SGK1 caused a further decrease in mitochondrial membrane potentials compared to HT22-NC-Dex group.

**Supplemental Figure 14**


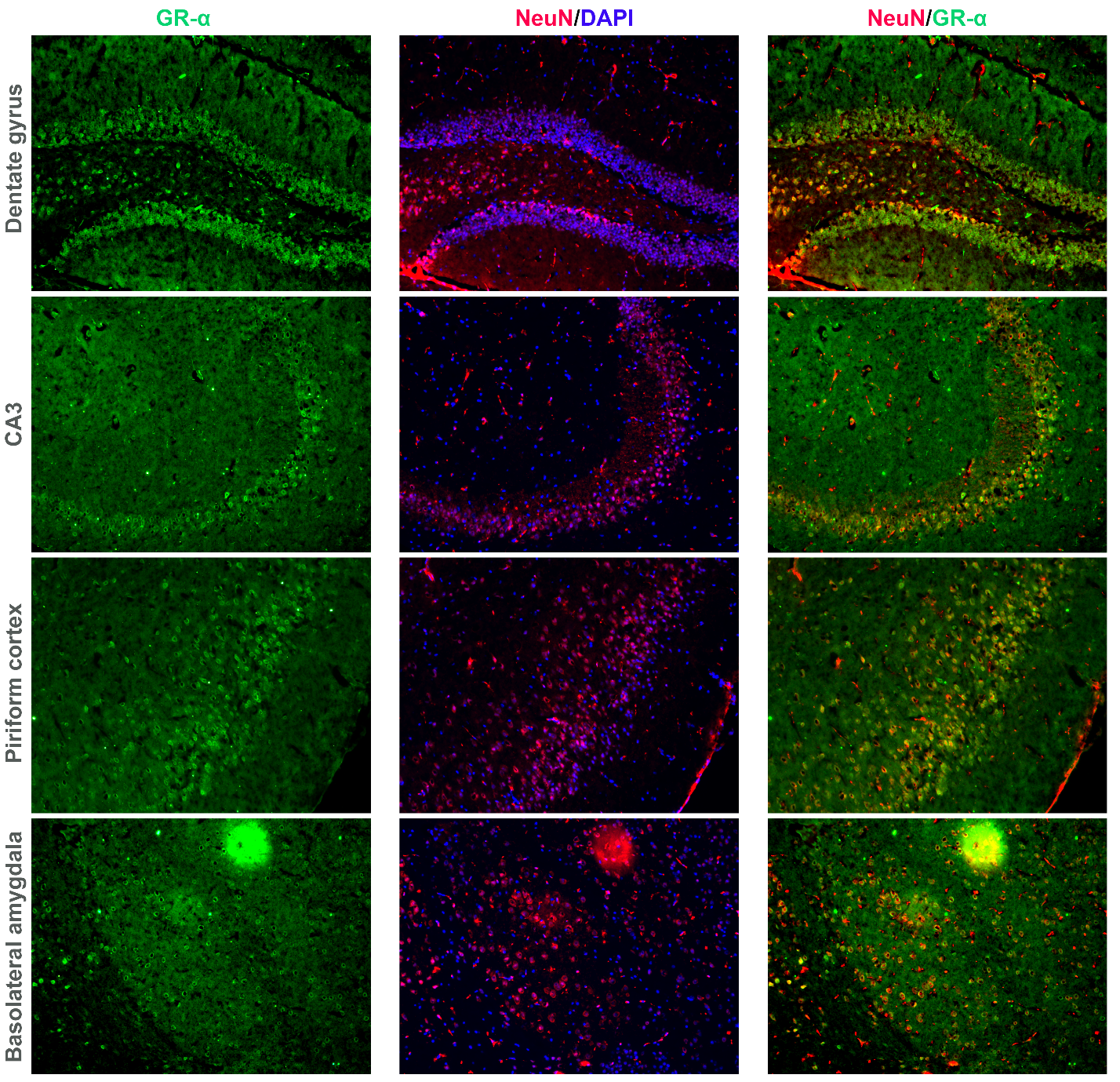


**Supplemental Fig. 14** Coronal sections of were costained with anti-NeuN (red) and anti-GRα (green). These immunofluorescences testified that GRα expression was widely distributed in mature neurons in various encephalic regions within the limbic nervous system.
