# Supplementary material for: SGK1 upregulation in GFAP+ neurons in the frontal association cortex protects against neuronal apoptosis after spinal cord injury
Source: Cell Death Dis. 2025 Apr 2;16(1):237. doi: 10.1038/s41419-025-07542-y (PMC11965300; doi:10.1038/s41419-025-07542-y)
Supplement: Supplementary file 2 — Uncropped IB blots [file 41419_2025_7542_MOESM2_ESM.pptx]

## Slide 1
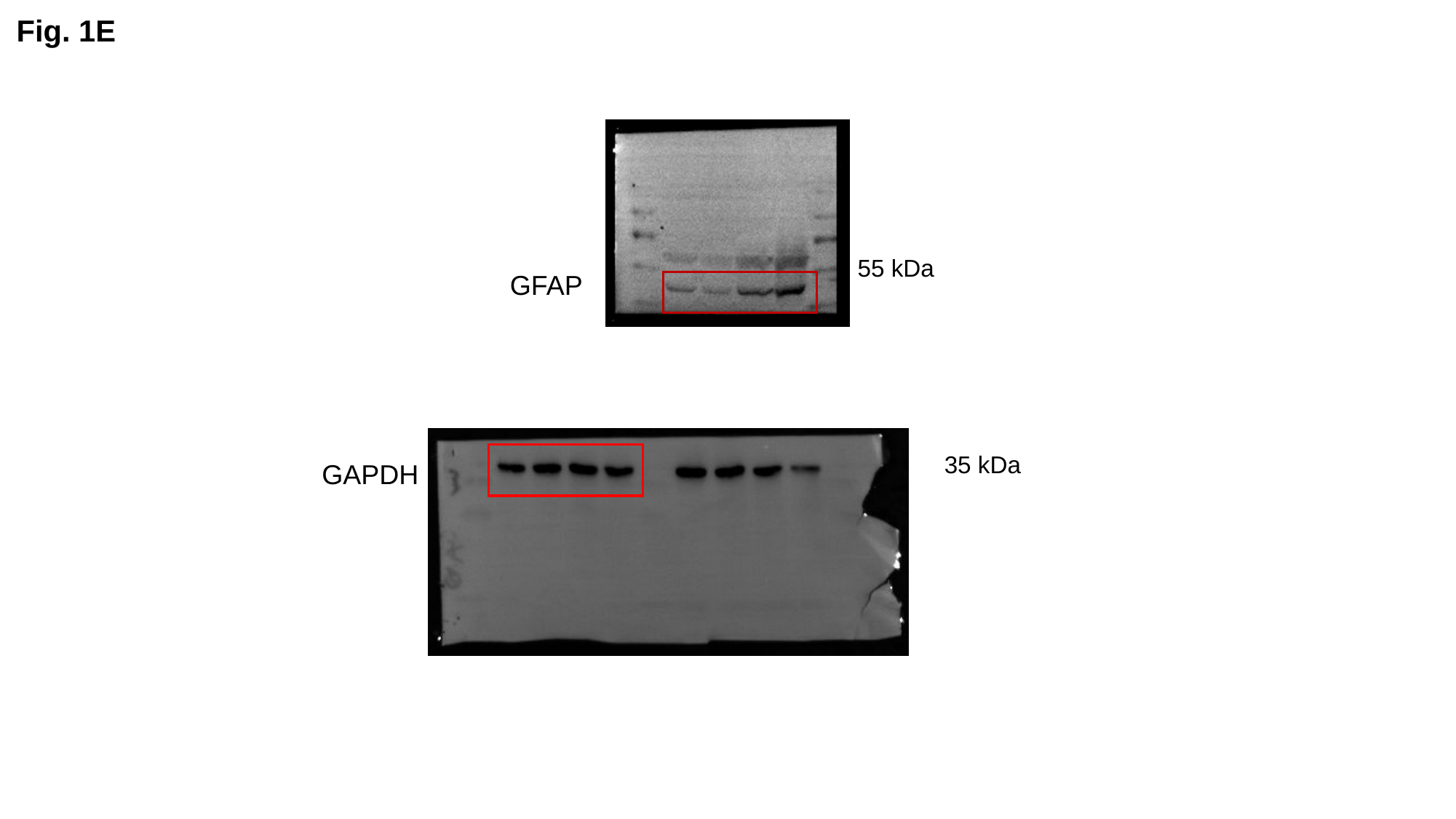

Fig. 1E
55 kDa
GFAP
35 kDa
GAPDH

## Slide 2
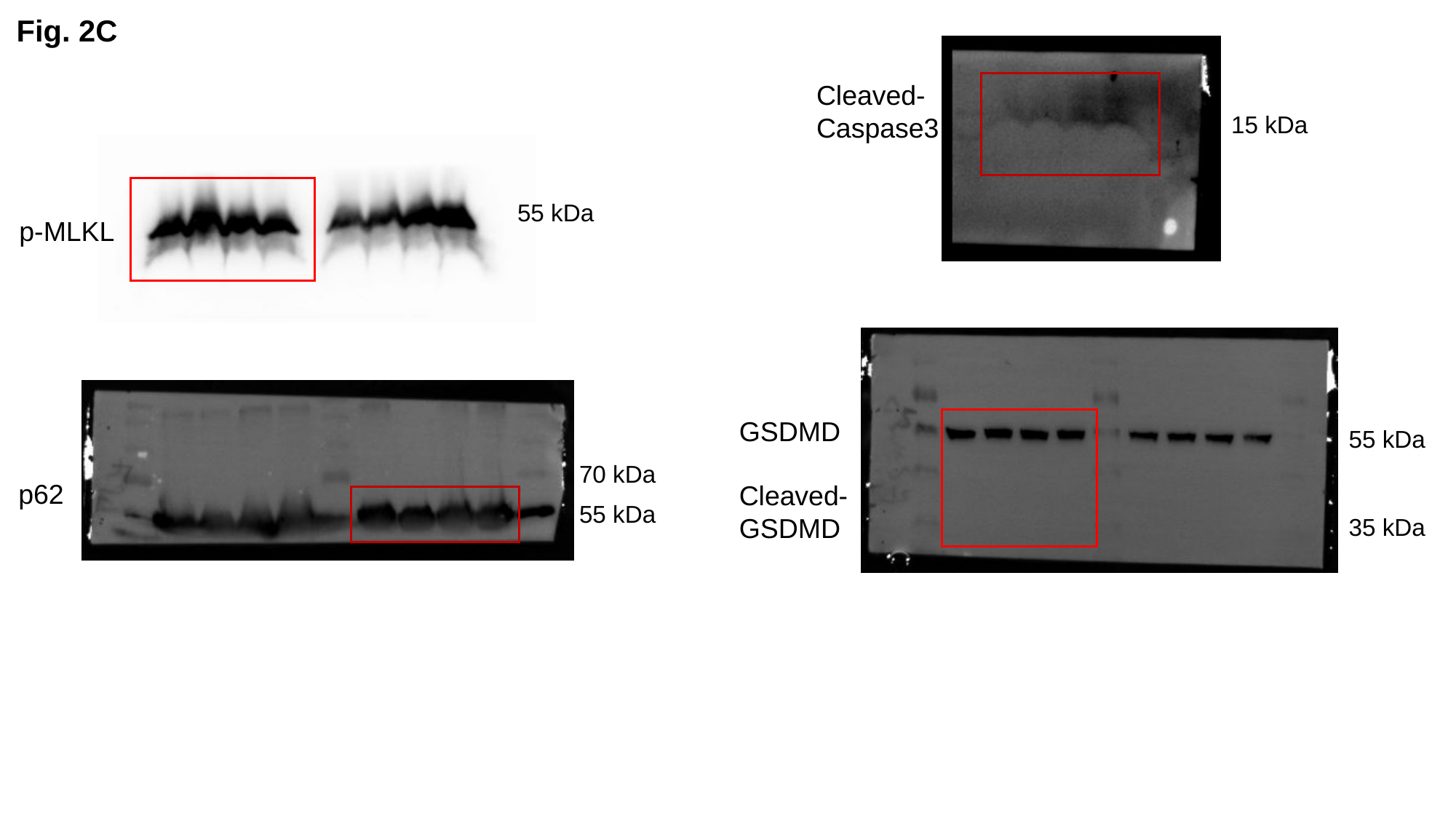

Fig. 2C
Cleaved-Caspase3
15 kDa
55 kDa
p-MLKL
GSDMD
55 kDa
70 kDa
p62
Cleaved-GSDMD
55 kDa
35 kDa

## Slide 3
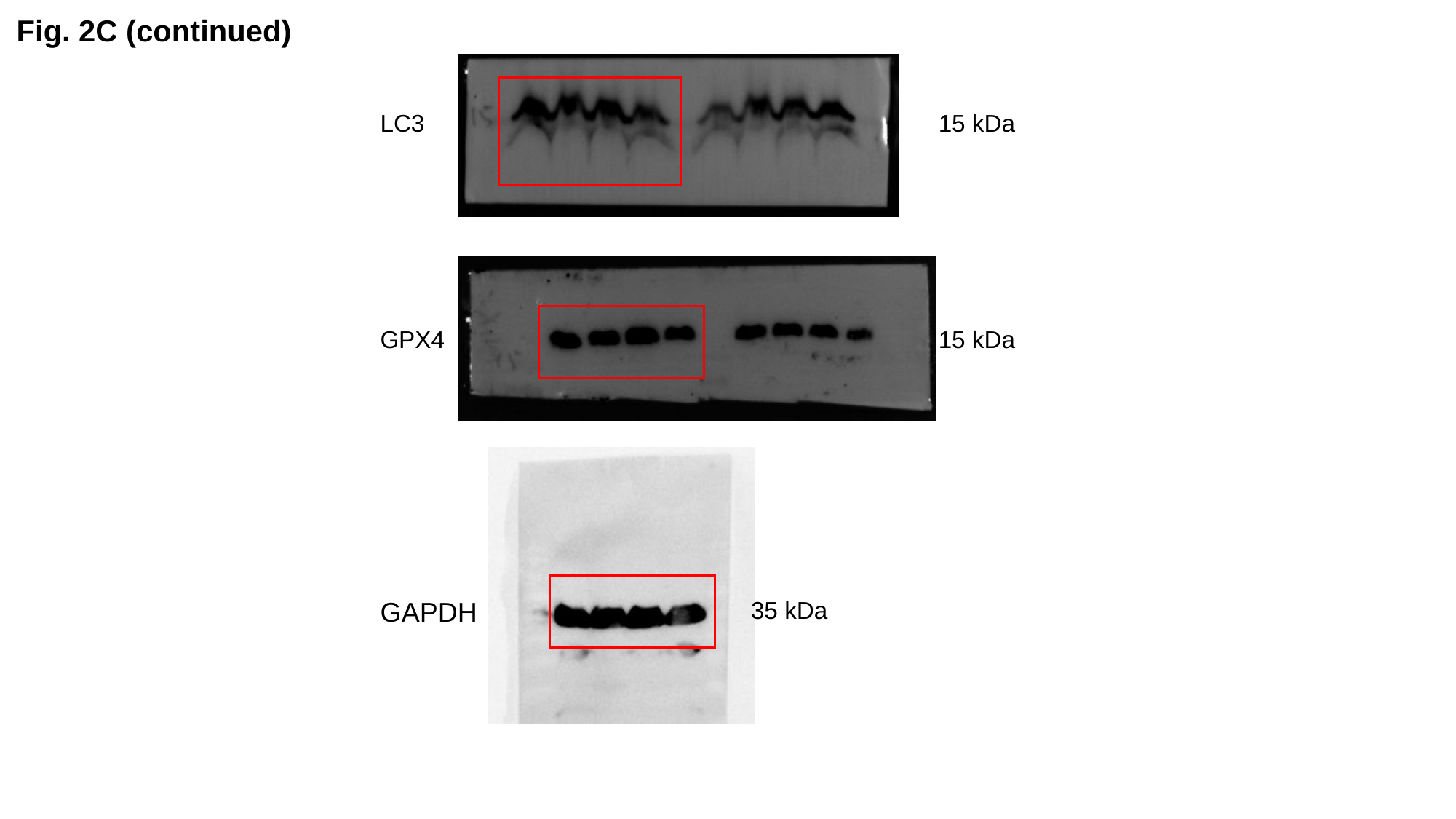

Fig. 2C (continued)
LC3
15 kDa
GPX4
15 kDa
GAPDH
35 kDa

## Slide 4
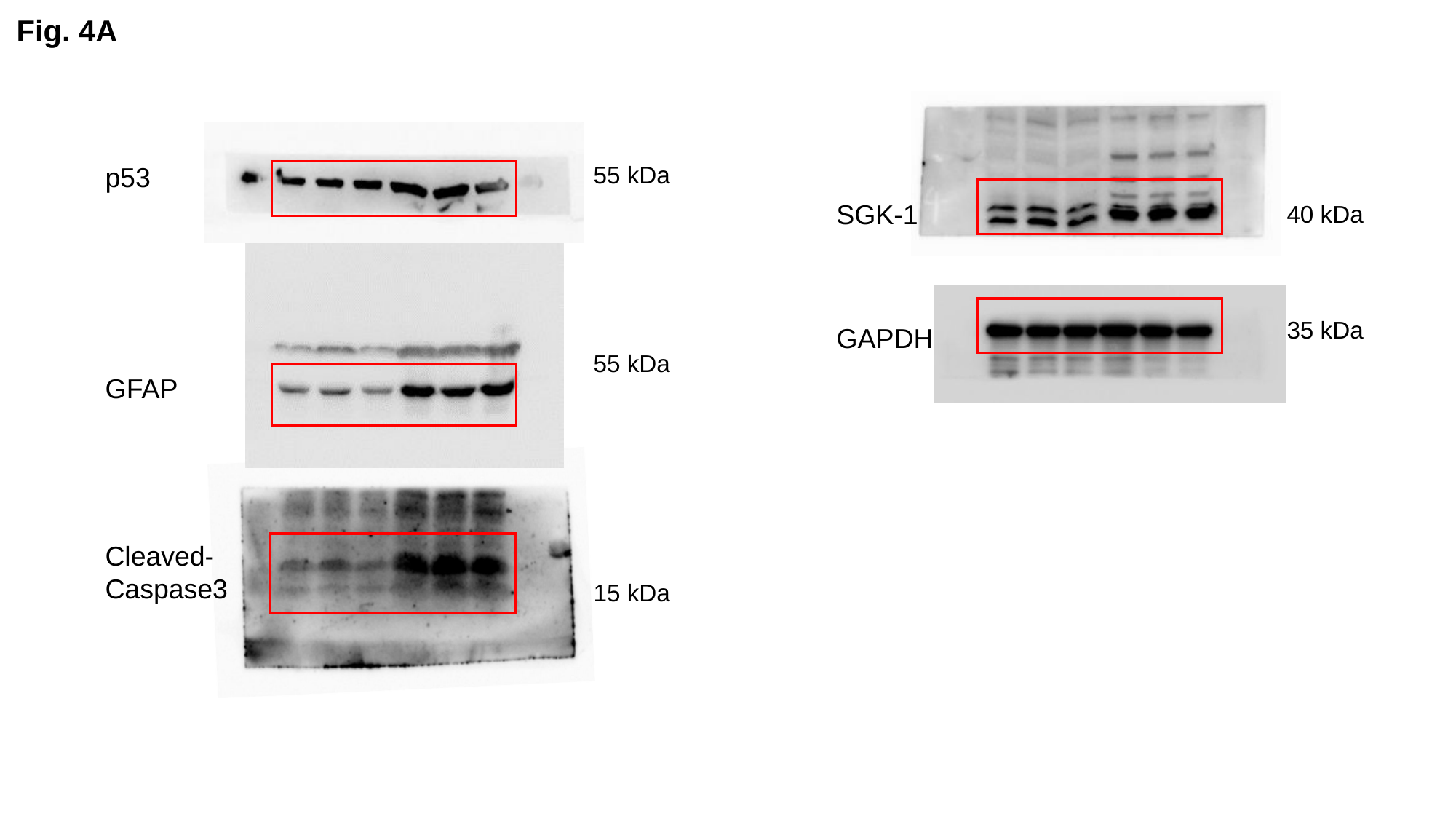

Fig. 4A
p53
55 kDa
SGK-1
40 kDa
35 kDa
GAPDH
55 kDa
GFAP
Cleaved-Caspase3
15 kDa

## Slide 5
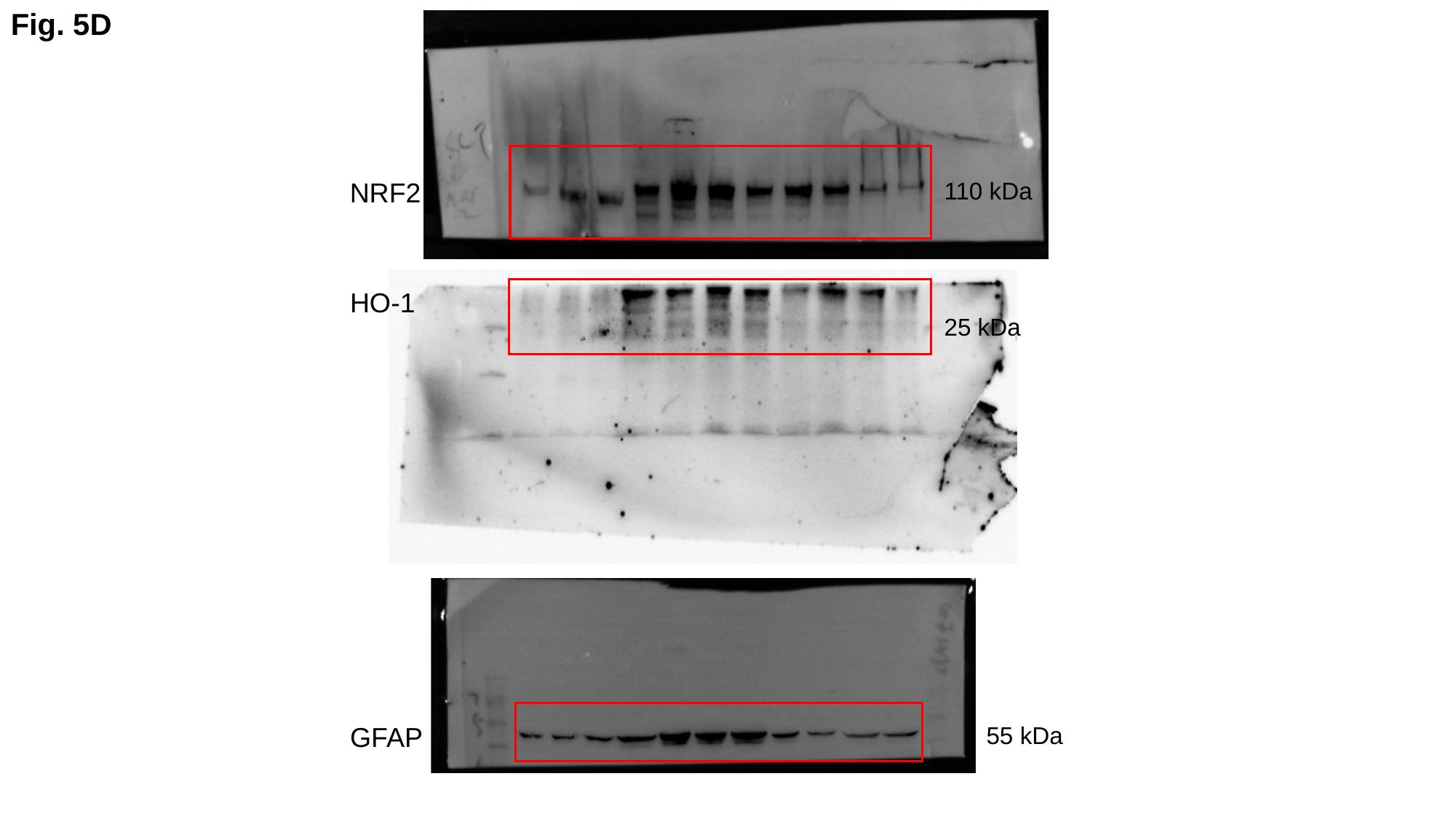

Fig. 5D
NRF2
110 kDa
HO-1
25 kDa
GFAP
55 kDa

## Slide 6
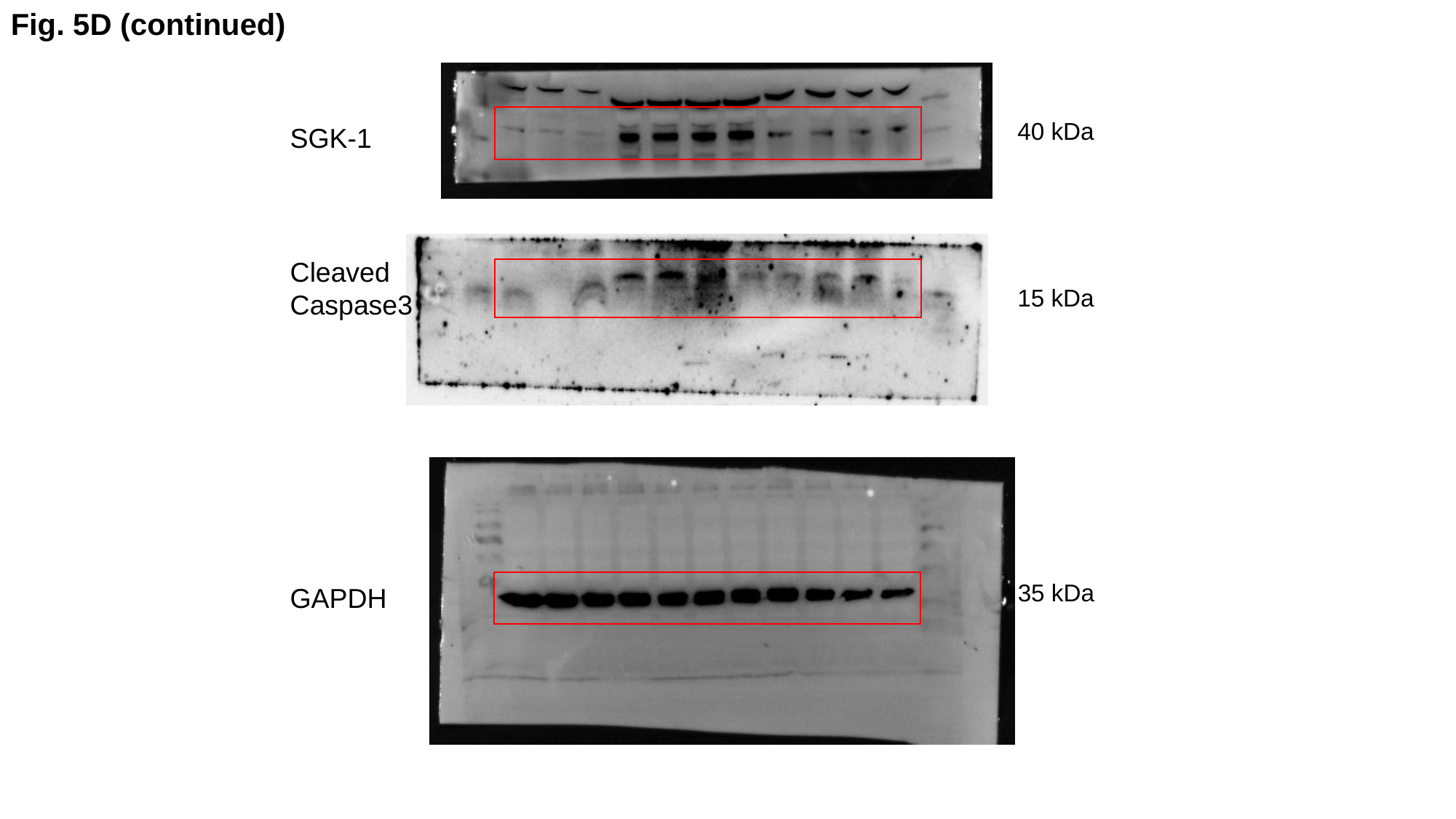

Fig. 5D (continued)
40 kDa
SGK-1
Cleaved
Caspase3
15 kDa
35 kDa
GAPDH

## Slide 7
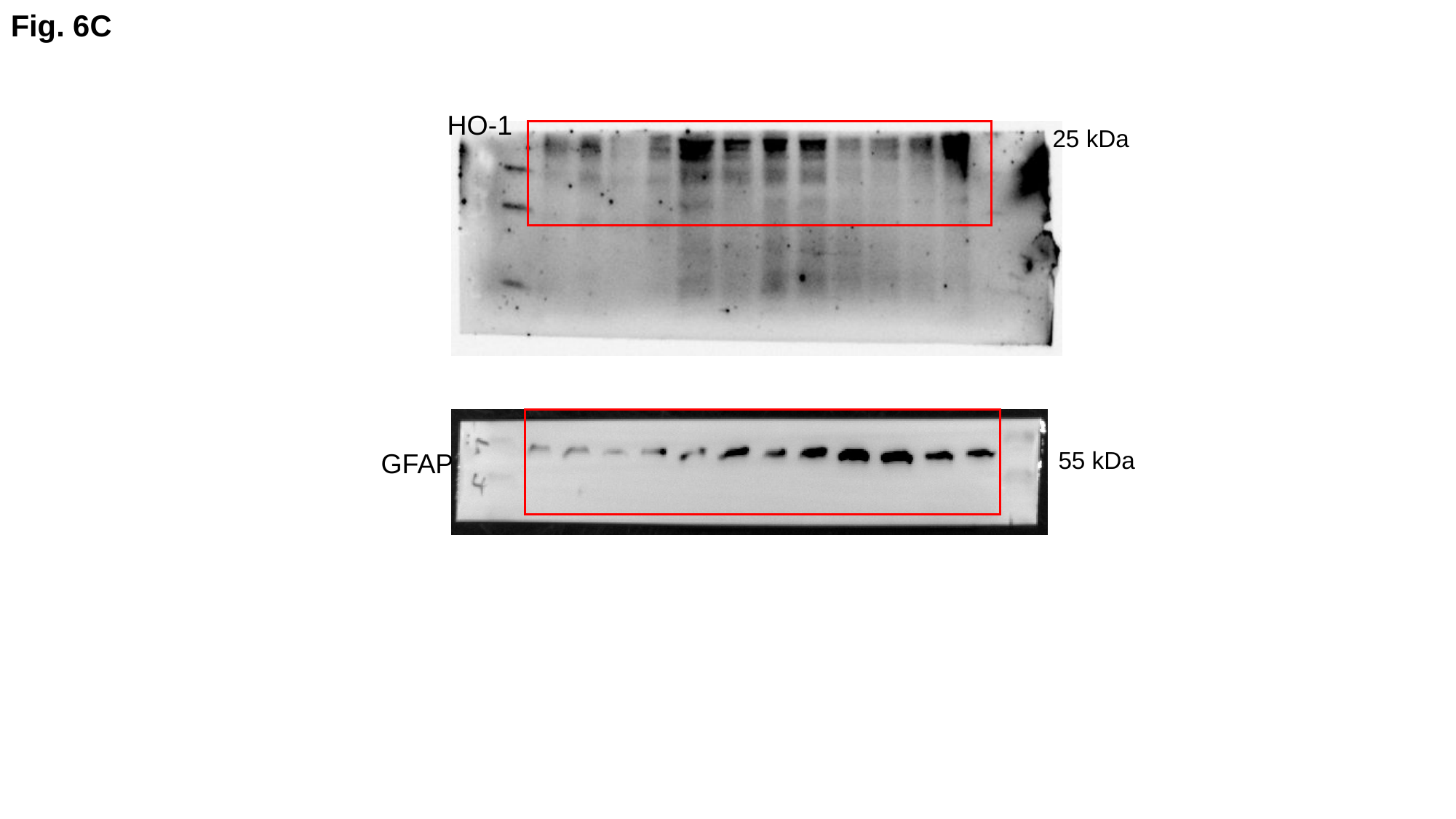

Fig. 6C
HO-1
25 kDa
55 kDa
GFAP

## Slide 8
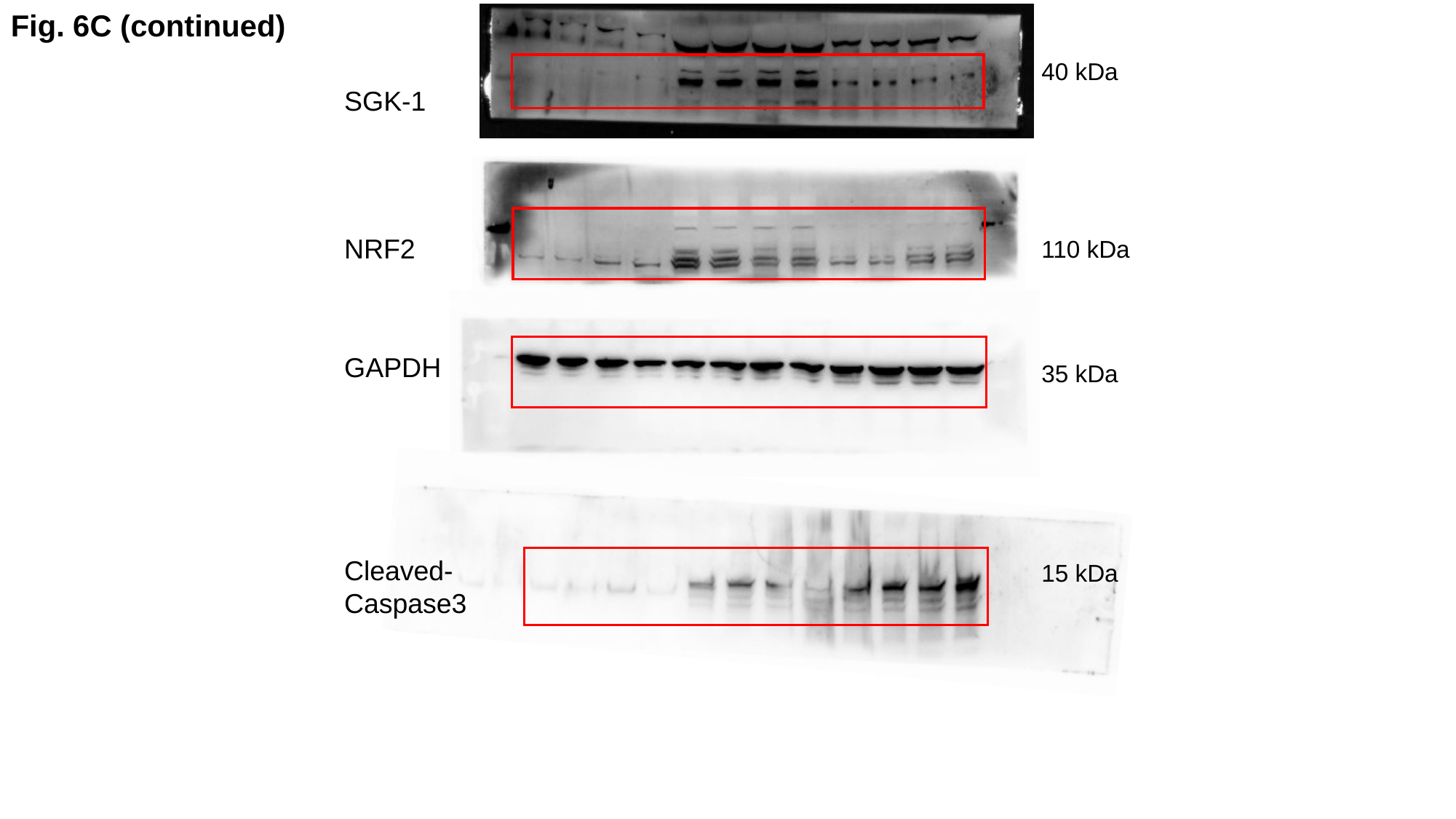

Fig. 6C (continued)
40 kDa
SGK-1
NRF2
110 kDa
GAPDH
35 kDa
Cleaved-Caspase3
15 kDa

## Slide 9
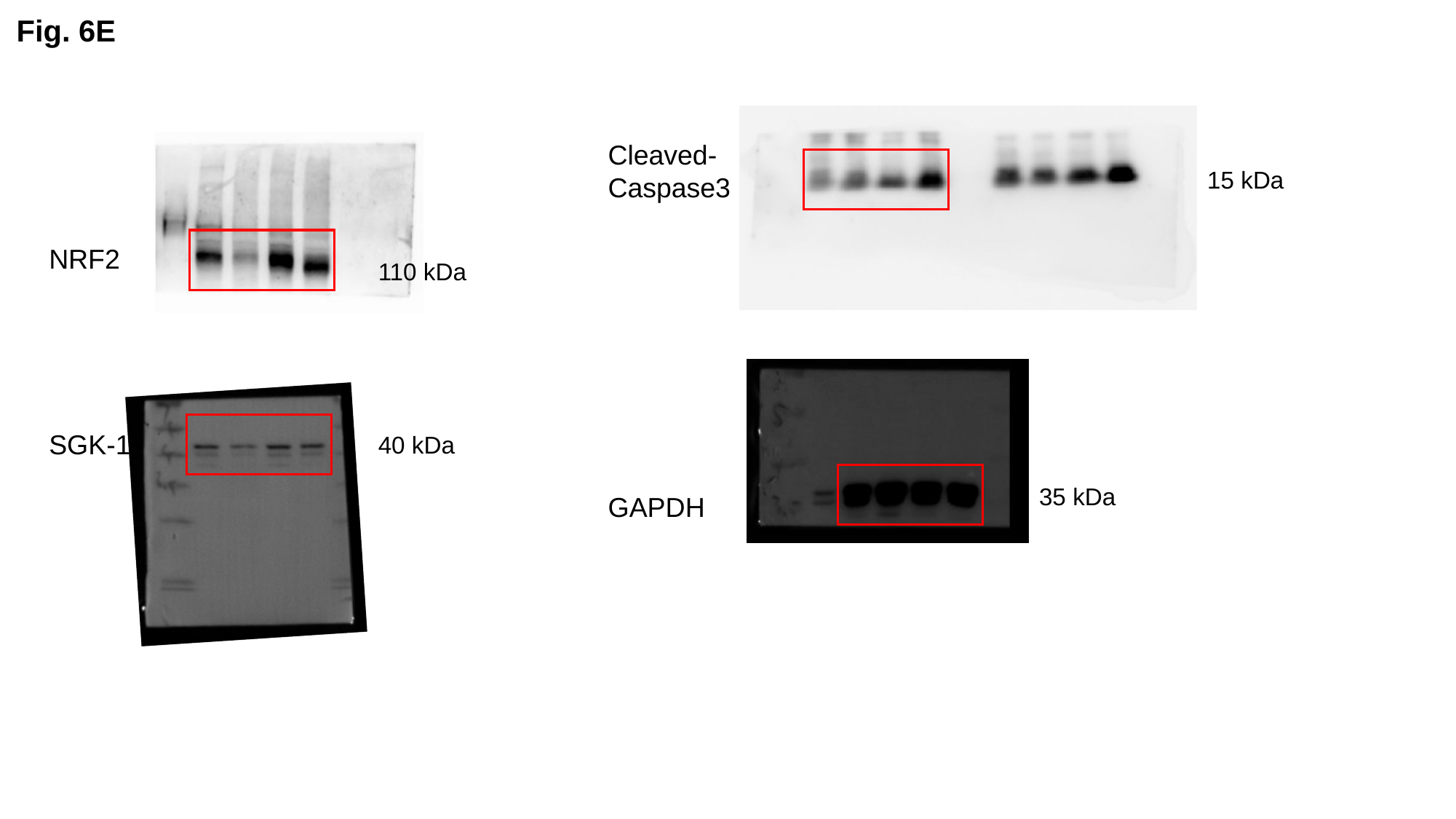

Fig. 6E
Cleaved-Caspase3
15 kDa
NRF2
110 kDa
SGK-1
40 kDa
35 kDa
GAPDH

## Slide 10
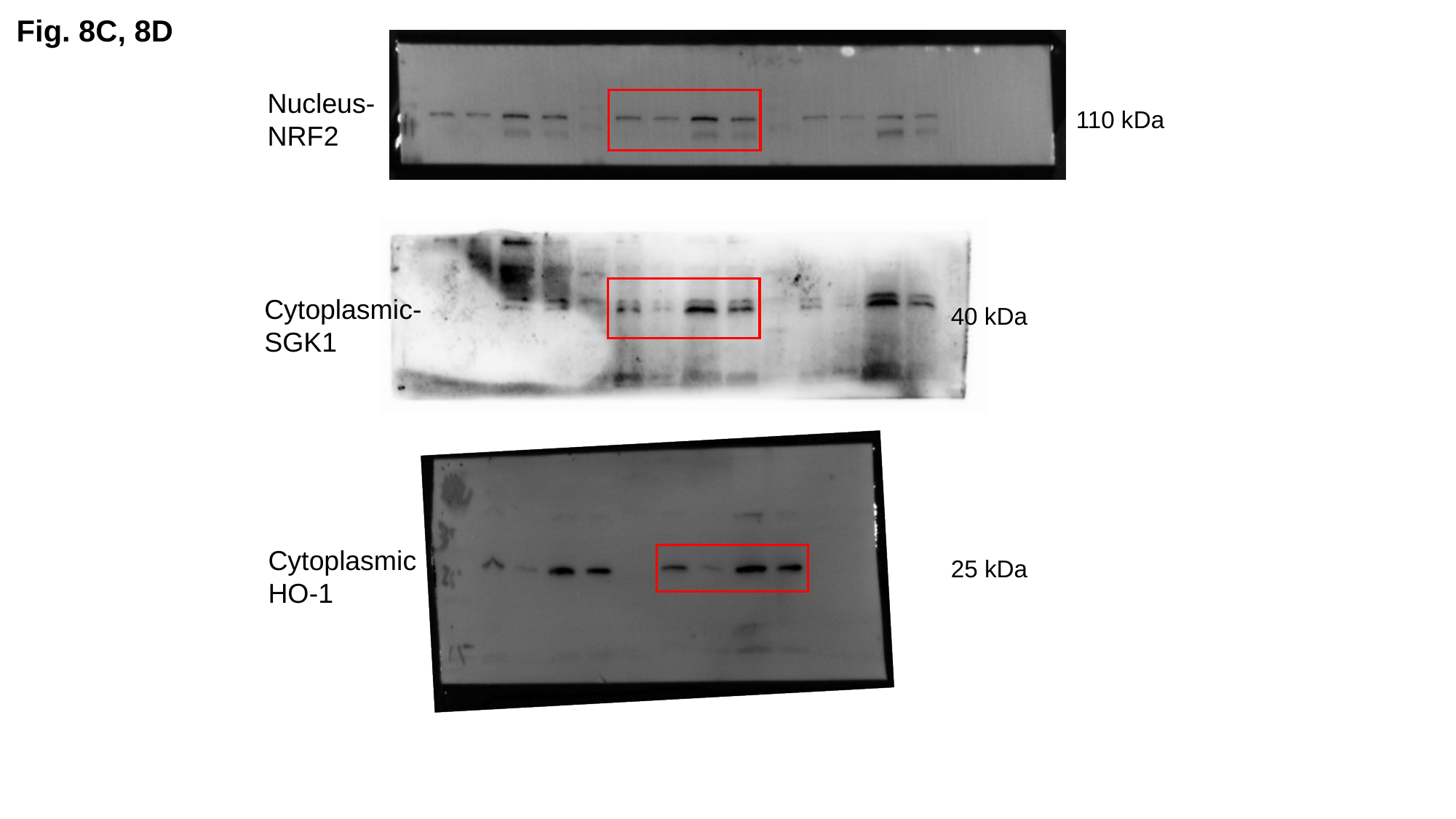

Fig. 8C, 8D
Nucleus-NRF2
110 kDa
Cytoplasmic-SGK1
40 kDa
Cytoplasmic
HO-1
25 kDa

## Slide 11
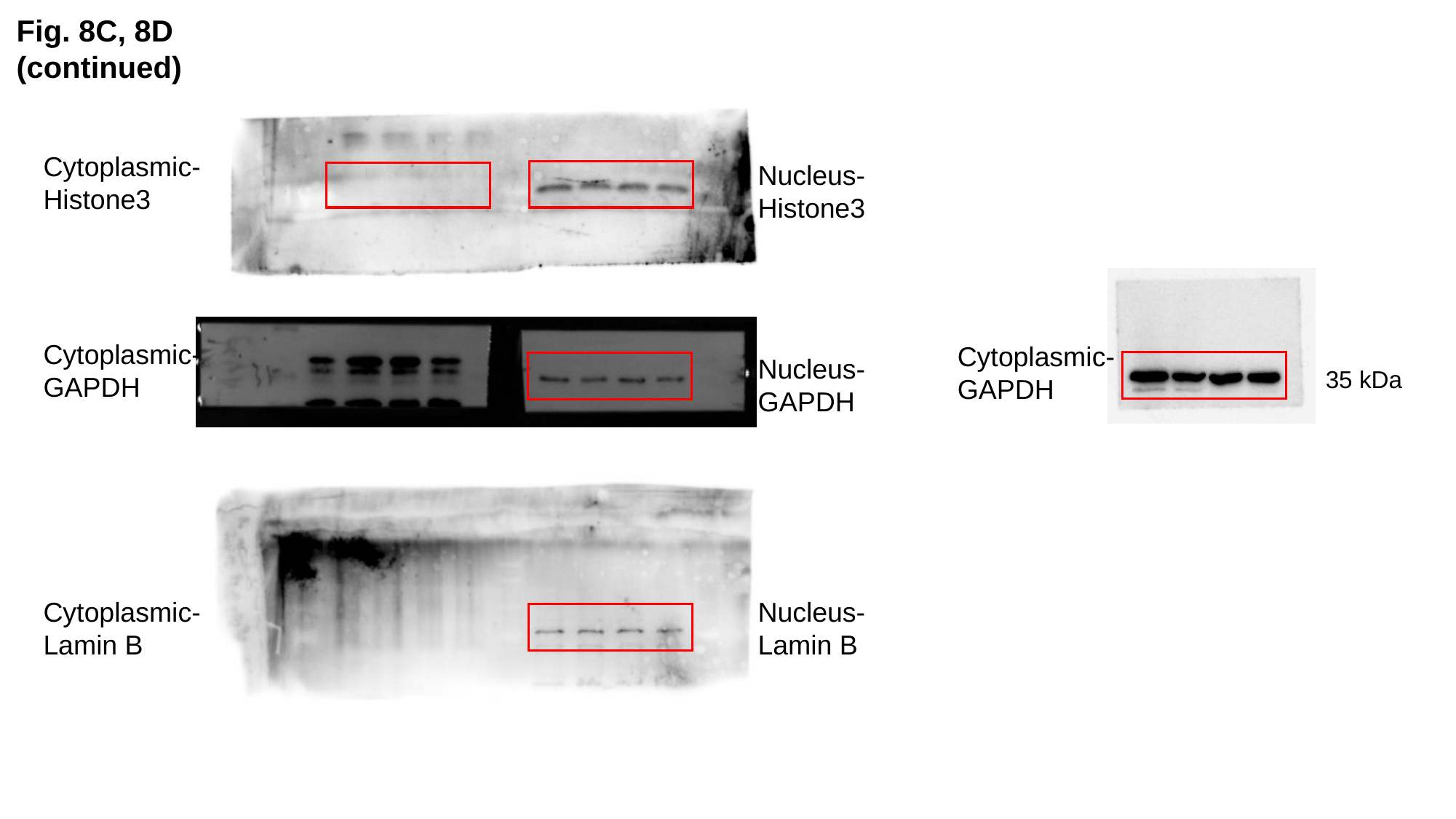

Fig. 8C, 8D (continued)
Cytoplasmic-
Histone3
Nucleus-
Histone3
Cytoplasmic-
GAPDH
Cytoplasmic-
GAPDH
Nucleus-
GAPDH
35 kDa
Cytoplasmic-
Lamin B
Nucleus-Lamin B

## Slide 12
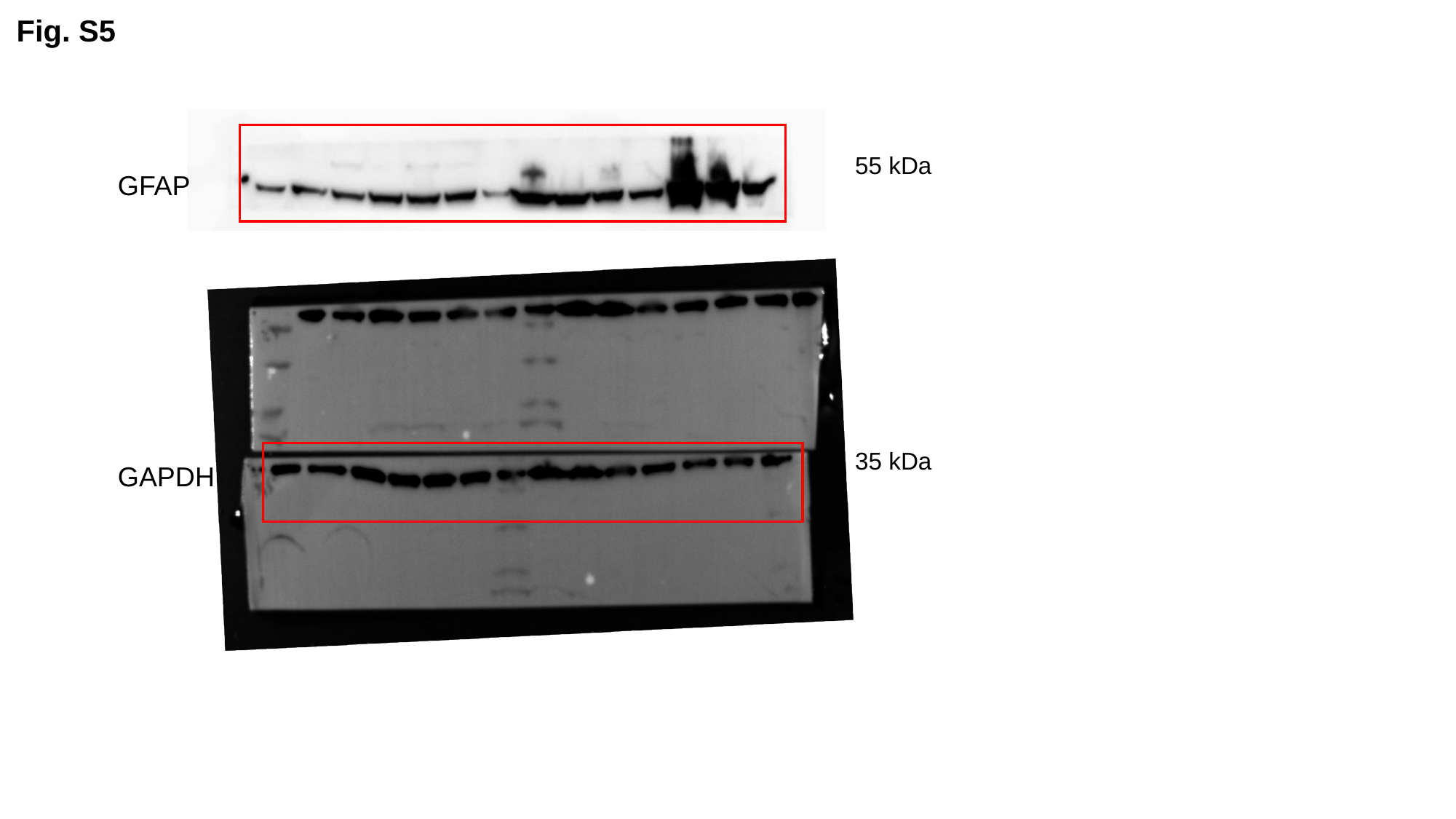

Fig. S5
55 kDa
GFAP
35 kDa
GAPDH

## Slide 13
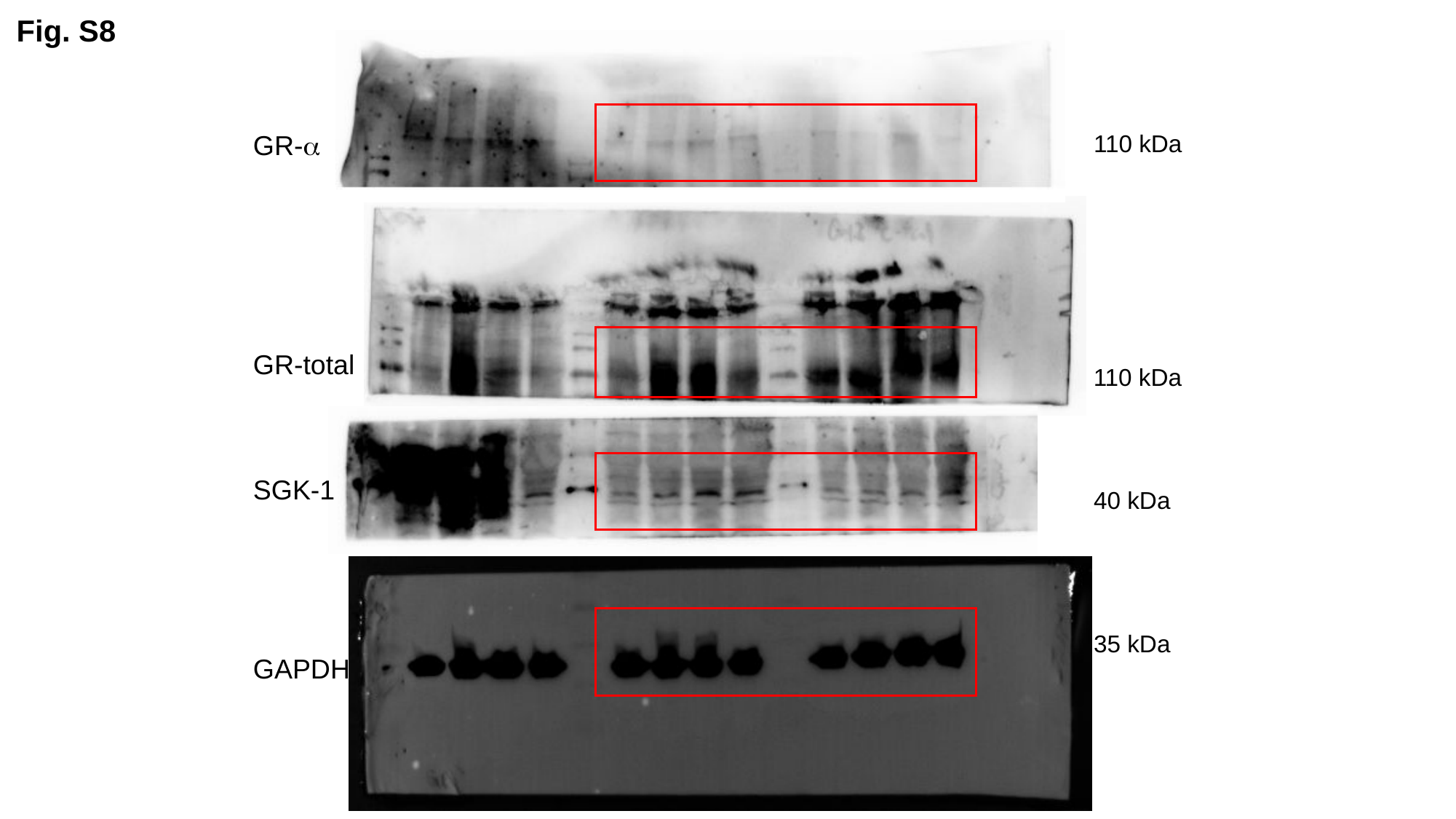

Fig. S8
GR-α
110 kDa
GR-total
110 kDa
SGK-1
40 kDa
35 kDa
GAPDH

## Slide 14
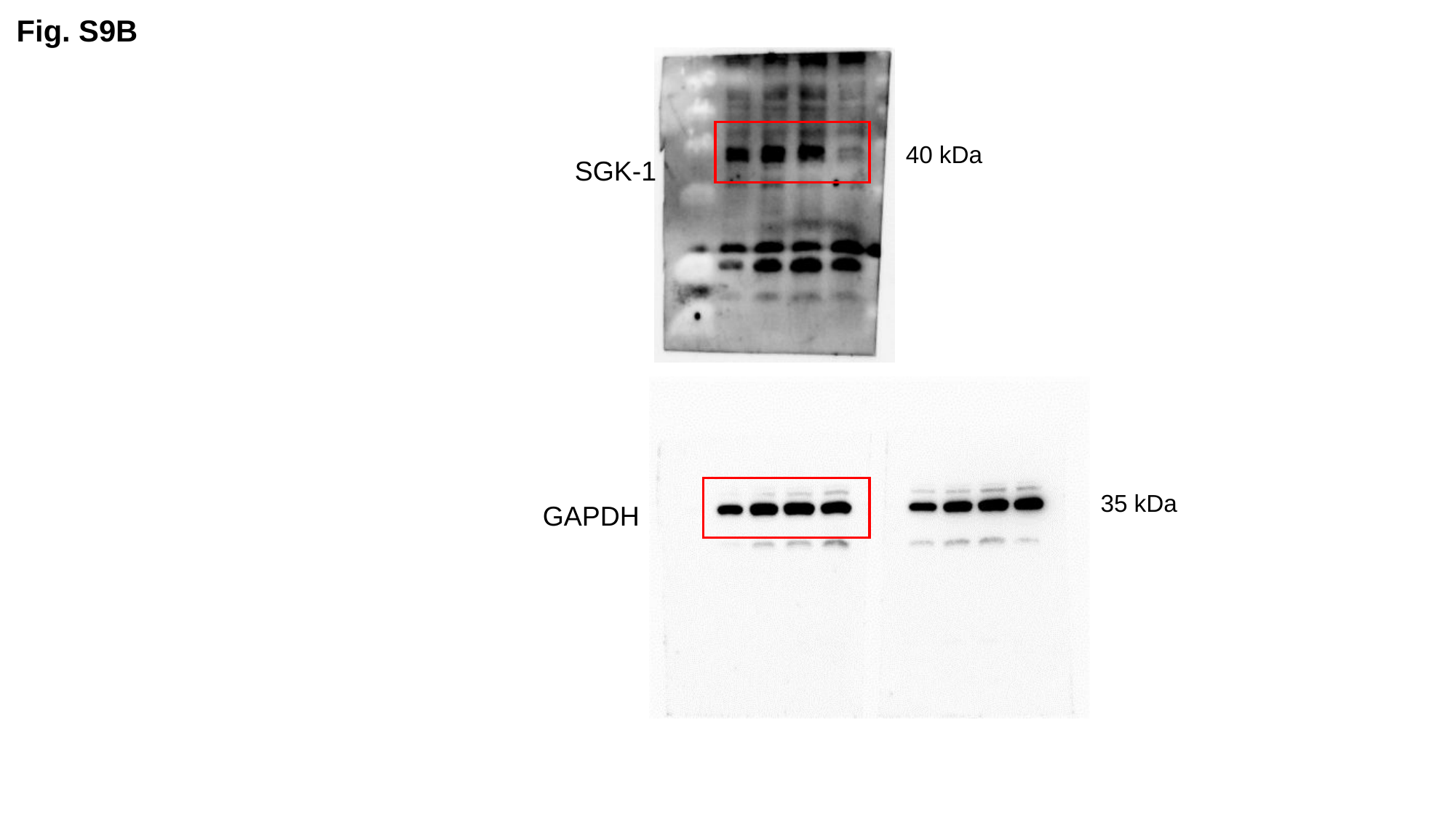

Fig. S9B
40 kDa
SGK-1
35 kDa
GAPDH
